# Predictability of the community‐function landscape in wine yeast ecosystems

**DOI:** 10.15252/msb.202311613

**Published:** 2023-08-07

**Authors:** Javier Ruiz, Miguel de Celis, Juan Diaz‐Colunga, Jean CC Vila, Belen Benitez‐Dominguez, Javier Vicente, Antonio Santos, Alvaro Sanchez, Ignacio Belda

**Affiliations:** ^1^ Department of Genetics, Physiology and Microbiology, Biology Faculty Complutense University of Madrid Madrid Spain; ^2^ Department of Microbial and Plant Biotechnology Centre for Biological Research (CIB‐CSIC) Madrid Spain; ^3^ Department of Soil, Plant and Environmental Quality Institute of Agricultural Sciences (ICA‐CSIC) Madrid Spain; ^4^ Department of Ecology & Evolutionary Biology Yale University New Haven CT USA; ^5^ Department of Microbial Biotechnology National Centre for Biotechnology (CNB‐CSIC) Madrid Spain; ^6^ Department of Biology Stanford University Stanford CA USA

**Keywords:** community‐function landscape, functional effect equations, microbial interactions, phylogenetic signal, wine yeasts, Evolution & Ecology, Microbiology, Virology & Host Pathogen Interaction

## Abstract

Predictively linking taxonomic composition and quantitative ecosystem functions is a major aspiration in microbial ecology, which must be resolved if we wish to engineer microbial consortia. Here, we have addressed this open question for an ecological function of major biotechnological relevance: alcoholic fermentation in wine yeast communities. By exhaustively phenotyping an extensive collection of naturally occurring wine yeast strains, we find that most ecologically and industrially relevant traits exhibit phylogenetic signal, allowing functional traits in wine yeast communities to be predicted from taxonomy. Furthermore, we demonstrate that the quantitative contributions of individual wine yeast strains to the function of complex communities followed simple quantitative rules. These regularities can be integrated to quantitatively predict the function of newly assembled consortia. Besides addressing theoretical questions in functional ecology, our results and methodologies can provide a blueprint for rationally managing microbial processes of biotechnological relevance.

## Graphical abstract

Exploring the connection between taxonomic composition and ecological function is a primary objective in engineering microbial consortia. Here, this link is investigated within the context of a highly relevant biotechnological process: wine fermentation.

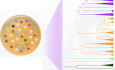

## Introduction

Microbes have been exploited for biotechnological purposes for millennia: from their original roles in food production to their myriad current uses in biomanufacturing. These microbial services are often delivered by complex and diverse ecological communities of microorganisms (Eng & Borenstein, 2019). Identifying the relationship between the taxonomic composition of microbial communities and the ecosystem functions they provide is critical if we wish to engineer microbial consortia and manage natural microbiomes (Dietze, 2018).

Ecological functions in complex communities emerge from individual contributions of each member species as well as from their ecological interactions. Thus, the first step to predictively linking taxonomy and ecosystem function is to determine whether the individual functional contributions of community members in isolation are in fact predictable from the phylogeny. This requires the existence of a phylogenetic signal in key functional traits, which is not always observed and, especially in microbes, cannot be taken for granted (Martiny *et al*, 2015). The second step is to learn how the functional contribution of each species will be altered by the presence of all other community members, that is to handle ecological interactions. Building predictive quantitative models of ecological interactions is a significant challenge (Friedman *et al*, 2017; Sanchez‐Gorostiaga *et al*, 2019; Gowda *et al*, 2022), as interactions can have complex mechanistic origins and their number explodes with community size.

The critical goal of predictively linking taxonomic composition and ecosystem function leads us to two key questions that we must resolve: (i) is there a phylogenetic signal in ecologically relevant traits, which determine the individual contributions of a species to community functions? and (ii) can we predict how the individual contributions of a species to a community‐function change in different community contexts, due to interactions with other community members?

As a model system to address these two questions, here we have studied wine fermentation—a system of profound socio‐economic significance and one of the first microbiomes ever domesticated and exploited by humans. In wine fermentation, the composition of the native yeast communities from grape musts (where a few dozens of yeast species can be found) changes over time as alcoholic fermentation proceeds. This ecological succession leads to the final dominance of *Saccharomyces cerevisiae*, which normally consume most of the available fermentable sugars in grape musts (approx. 200 g/l of glucose:fructose, in 1:1 ratio). Despite the complexity of this yeast species succession, wine fermentation is a reproducible process; grape must harbours a moderate number of different yeast species that can be easily isolated and assembled in synthetic grape must to create synthetic yeast communities, and some basic functions of wine microbial communities can be easily measured by handling methods such as biomass production or sugar consumption quantifications. Wine fermentation is therefore emerging as a model system for synthetic ecology (Boynton & Greig, 2016; Bagheri *et al*, 2020; Conacher *et al*, 2020, 2022; Belda *et al*, 2021; Jahn *et al*, 2023).

To address the two questions outlined above in the wine fermentation system, here we first interrogate whether key functional traits are predictable from the phylogeny. To this end we characterise the phylo‐functional relationships among wine yeast species, measuring a total of 43 ecological and industrial traits in a collection of 60 wine yeast strains belonging to 30 different species (spanning the expectable phylogenetic diversity of most commonly found wine yeasts). We find that the environmental preferences of yeasts, and to a lesser extent their metabolite contribution to wine fermentations, exhibit phylogenetic signal and can be imputed from a standard fungal genetic marker (26S sequence). We then show that the contributions of individual wine yeasts to alcoholic fermentation can be predicted in different community contexts, as they follow simple quantitative rules that parallel the global epistasis concept in evolutionary genetics. Using methods that we have introduced in recent work (preprint: Diaz‐Colunga *et al*, 2023) to infer the community‐function landscape from a subset of observed consortia, we are able to quantitatively predict the wine fermentation function in complex wine yeast consortia, based on their community composition.

## Results

### A strong phylogenetic signal allows to predict relevant traits in wine yeasts

**Figure 1:**
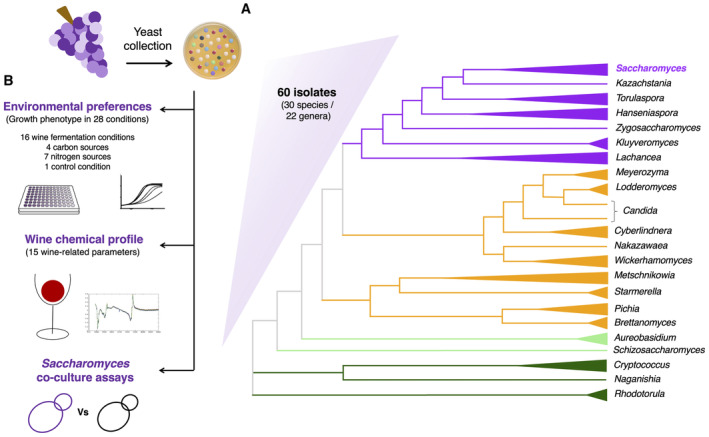
Composition and phenotyping strategy of the wine yeasts collection Schematic representation of the phylogenetic relationships of the 22 yeast genera included in the wine yeasts collection studied (based on the maximum likelihood phylogenetic tree presented in Appendix Fig S1, made using the partial 26S nucleotide sequence of the 60 strains of the study). Colours of the branches correspond to the lowest taxonomic level shared with *S. cerevisiae* (purple: same family, orange: same order, light green: same division, dark green: same kingdom). Triangles at the end of the branches represent the different genera of the yeast collection. The length of the triangles refers to the distance from the common ancestor of each strain belonging to the different genera.We explored the most relevant ecological and industrial (enological) traits of the 60 wine yeast strains included in our collection, by characterising: (i) their environmental preferences, as their growth efficiency in a panel of 28 conditions (detailed in Table EV1), including 16 wine fermentation conditions (modulating the sugar concentration, pH, antimicrobials, temperature, nitrogen or vitamins availability in synthetic grape must (SGM)), four carbon sources (the three main organic acids found in the grape must, and glucose, as sole carbon sources) and seven nitrogen sources (the six most abundant amino acids found in grape must, and ammonia, as sole nitrogen sources), and a synthetic medium as a growth control condition; (ii) their fermentation performance, measuring 15 physicochemical parameters in wines (detailed in Table EV2) such as sugar consumption, ethanol production, organic acids production/consumption or total acidity produced, in laboratory‐scale fermentations of SGM; and (iii) their effect on the growth efficiency of co‐cultures with *S. cerevisiae* (non‐*Saccharomyces* × *S. cerevisiae*) in SGM.

To address our first question—is there a phylogenetic signal for ecological and industrial traits within the wine yeast community—it is necessary to assay a broad collection of yeasts covering the widest possible phylogenetic diversity within the wine yeast microbiome. Thus, we established a collection of 60 wine yeast strains (Dataset EV1), including 30 different species (belonging to 22 genera and 10 families; both Ascomycota and Basidiomycota yeasts; Fig 1A and Appendix Fig S1). All the yeast strains in our collection were isolated from wine environments (see details about geographical origin and year of isolation in Dataset EV1), and it covers genera that are cosmopolitan (i.e. *Saccharomyces*, *Aureobasidium*, *Hanseniaspora*, *Metschnikowia* or *Lachancea*) as well as rare (*Cyberlindnera*, *Kluyveromyces* or *Brettanomyces*) in wine fermentations (Appendix Fig S2). We then phenotyped this collection, focussing on traits that are relevant from an ecological and industrial (enological) standpoint (Fig 1B). Hence, we measured the growth ability of the strains in a panel of 28 wine‐related culture conditions (Table EV1) to infer their environmental preferences, and we evaluated the fermentation performance of the strains by studying their contribution to the chemical profile (measuring 15 physicochemical parameters, Table EV2) of the resulting wines produced by each strain alone in laboratory‐scale fermentations. To our knowledge, this represents the broadest functional catalogue of wine yeast species currently available (Dataset EV2).

**Figure 2:**
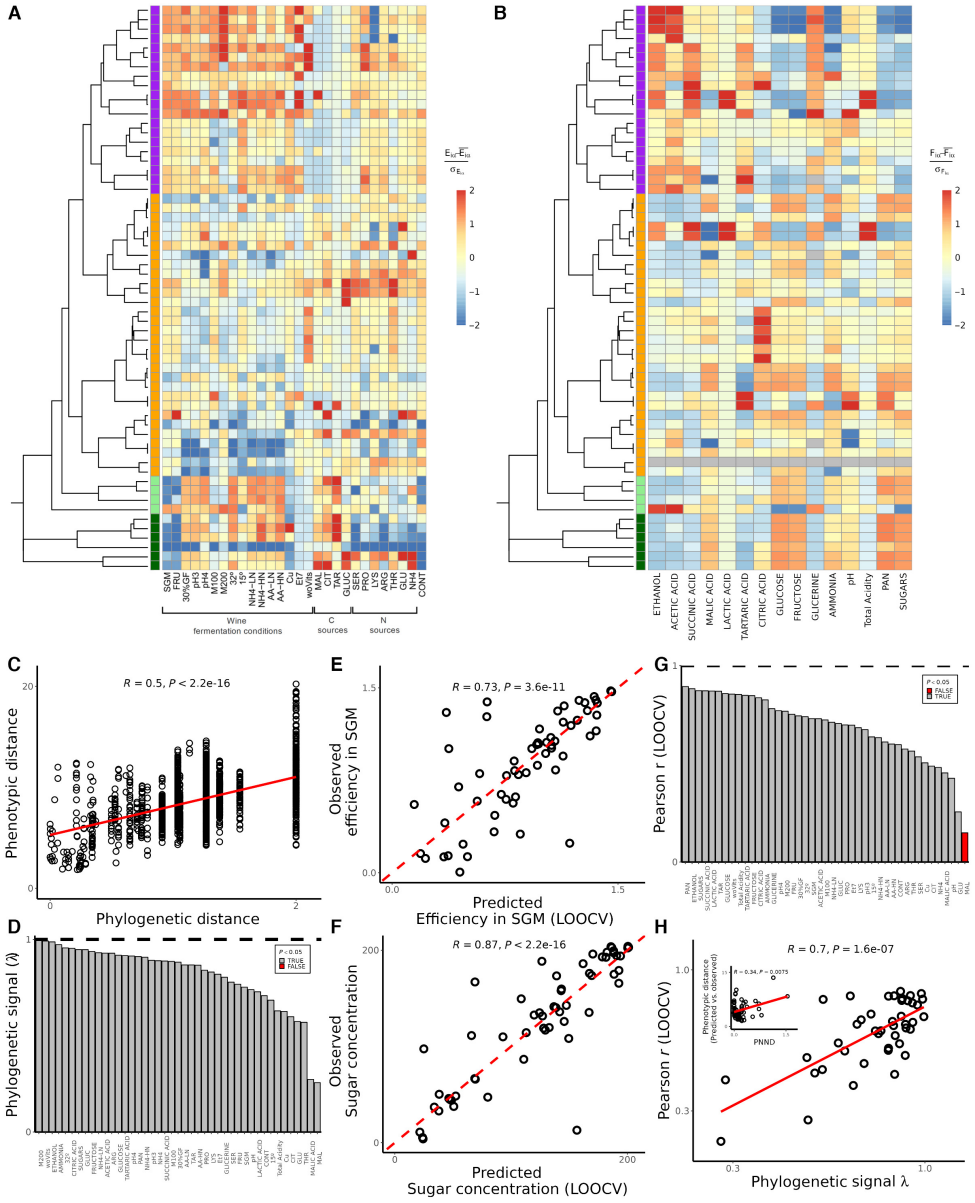
Environmental preferences and metabolite production of yeasts in wine fermentations can be predicted from phylogeny A, BPhylogenetic tree and trait heatmaps for 60 wine yeast strains showing (A) their environmental preferences, as growth efficiency in 28 different growing conditions (see Table EV1 for detailed information about conditions tested), and (B) their wine fermentation performance, measuring 15 wine physicochemical parameters after 168 h of fermentation in synthetic grape must (SGM; see Table EV2 for detailed information about the parameters measured). *Brettanomyces bruxellensis* Bb_1 strain is removed from this analysis as it was unable to grow under this fermentation assay (grey row in the heatmap). Dataset EV2 contains the raw data used to plot the heatmaps. Trait values are centred and scaled for visualisation purposes. Colours to the left of the heatmap correspond to the lowest taxonomic level shared with *S. cerevisiae* (purple: same family, orange: same order, light green: same division, dark green: same kingdom). Traits heatmap with the strains clustered based on the phenotypic data are shown in Appendix Fig S4A and B.CWe find a significant relationship between pairwise phylogenetic (cophenetic) distance and pairwise phenotypic distance (Euclidean distance in scaled trait values).DPhylogenetic signal (Pagel's λ, hereafter λ) for all 43 traits measured (environmental preferences and fermentation performance). The λ parameter represents a continuous measure that varies from 0 to 1. λ = 0 indicates that the trait has evolved independently of the phylogeny whereas λ = 1 indicates that the trait distribution among strains is highly influenced by their shared evolutionary history. 41/43 traits show a significant phylogenetic signal.EPredicted vs. observed growth efficiency in SGM.FPredicted vs. observed sugar (glucose + fructose) concentration after 168 h of growth in SGM.GCorrelation coefficient between predicted and observed trait values for all 43 traits. A significant positive correlation is observed for 41/43 traits (Appendix Fig S5).HTraits with stronger phylogenetic signals are better predicted using phylogenetic imputation. Furthermore, strains with a greater phylogenetic nearest neighbour distance (PNND), indicating a larger phylogenetic distance between them, tend to exhibit less accurate predictions for their phenotypic traits (*R* = 0.55; *P* = 6.3e‐6). Data information: All predictions (panels E and F) were made using phylogenetic imputation (Rphylopars) and tested using LOOCV. Source data are available online for this figure.

This extensive functional characterisation revealed a large phenotypic diversity across wine yeasts (Fig 2A and B). A first look at the heatmaps suggests a qualitative connection between the functional traits of the strains and their phylogenetic relationships, as we observe some distinguishable phenotypic patterns across taxonomic clusters both for environmental preferences (Fig 2A) and fermentation performance (Fig 2B), also observed in Appendix Fig S4A and B. For instance, in wine fermentation conditions, Saccharomycetaceae strains (purple) have, on average, higher growth efficiencies (total variation in cellular density) than the non‐Saccharomycetaceae strains (orange and greens; *t*‐test, *P* = 1.604e‐12), and, as expected at the species level, *S. cerevisiae* strains showed the highest growth efficiency in most fermentative conditions tested (Fig 2A) and it appeared as the only species able to complete the wine fermentations (consuming more than 95% of the initial glucose + fructose content) in the laboratory‐scale fermentations performed in synthetic grape must (SGM; Fig 2B).

In order to quantify the relationship between phylogeny and phenotype, we next took advantage of the 26S sequence of the 60 yeast strains phenotyped (Dataset EV1) as well as an additional set of 53 strains not included in our phenotypic assays (Table EV3). We constructed a maximum likelihood phylogenetic tree for our entire collection of 113 strains (Appendix Fig S7) and used this tree to quantify the phylogenetic (cophenetic) distance between every pair of yeast strains. We then determined the phenotypic similarity between every pair of 60 strains, as the Euclidean distance between the scaled trait values, both from the environmental preferences and the fermentation performance of the studied strains. We find that there is a significant correlation (*R* = 0.5, *P* = 2.2e‐16) between the phylogenetic distance (branch length) and phenotypic distance (Euclidean distance in trait space) between pairs of strains (Fig 2C). This correlation is much stronger for the environmental preferences phenotype (*R* = 0.58, *P* = 2.2e‐16; Appendix Fig S3A) than for the metabolite contribution of yeasts to wine fermentations (*R* = 0.089, *P* = 0.00024; Appendix Fig S3B). Furthermore, all phenotypic traits analysed in this work exhibited significant phylogenetic signal (Pagel λ, *P* < 0.05 based on 1,000 permutations; see Materials and Methods; Fig 2D). At this point, we wondered whether the phylogenetic signal observed across wine yeast strains would allow us to quantitatively predict unobserved trait values in strains based on their phylogenetic position. To test this hypothesis, we leveraged *Rphylopars*, a tool for phylogenetic imputation of missing data (Goolsby *et al*, 2017). *Rphylopars* imputes phenotypic values in unobserved species by rerooting the phylogeny at the most recent common ancestor of the taxon with unobserved traits and the rest of the tree and then performing ancestral state reconstruction to obtain a maximum likelihood estimate for the trait value in this common ancestor (in this case under a Brownian model of trait evolution; Bruggeman *et al*, 2009). The ability to predict unobserved trait values using phylogenetic imputation was determined by performing Leave One Out Cross‐Validation (LOOCV) method and then comparing observed trait values with those predicted by *Rphylopars*. In Fig 2E and F, we show a significant correlation between predicted and observed values for two different traits, growth efficiency (*R* = 0.73, *P* = 3.6e‐11) and sugar consumption in SGM (*R* = 0.87, *P* < 2.2e‐16). Similar correlations are observed for most of the other 41 traits measured in this study (Appendix Fig S5), with all but one trait showing significant Pearson correlation coefficients between predicted and observed trait values (Fig 2G).

Interestingly, we found that we were unable to predict growth on malic acid as the sole carbon source, which was consistent with it being the trait with the lowest phylogenetic signal (λ = 0.25, Fig 2D). More generally and consistent with simulation studies (Goberna & Verdú, 2016), in our empirical dataset, traits with a stronger phylogenetic signal (e.g. total acidity and ethanol production in SGM fermentations) showed a better predictability using phylogenetic imputation (Fig 2H). Furthermore, predictions were better for strains where a close relative was present in the tree (low phylogenetic nearest neighbour distance, PNND) that could be leveraged when imputing trait values (inset in Fig 2H). To check the accuracy of phylogenetically based functional trait predictions out of sample, we phenotyped 53 new strains (Table EV3, Appendix Fig S7) measuring their growth efficiency in SGM (Table EV4), and the original 60 strain library was assayed again for this phenotype as a control. Using the phylogenetic imputation method trained with the original dataset of our 60 strains collection, we show that it is possible to predict the growth phenotype of 53 new strains, never tested before—by using the phylogeny imputation described here—with a similar accuracy as replicate measurements of the training set of strains in this independent experiments (Appendix Fig S8).

### Mapping the community‐function landscape allows to predict the function of complex yeast communities

*Saccharomyces cerevisiae* is the dominant yeast in wine fermentations, and it is able to complete the alcoholic fermentation process on its own, depleting at least 95% of the fermentable sugars of grape musts. However, its ecological partners (non‐*Saccharomyces* yeasts) could also play an important role in wine ecosystem functioning, either through direct contributions to the fermentation process, through interactions with *S. cerevisiae*, or both. Importantly, the contribution of each of these yeasts to the wine fermentation function is likely to depend on interactions with the other community members. Mapping out these interactions is essential if we wish to quantitatively predict how the composition of the yeast community affects the function of the wine ecosystem. To explore this question, we would have to place these wine yeasts in different community contexts and determine how their contribution to the sugar consumption (as the main function of the wine ecosystem) varies as the composition of the community changes.

**Figure 3:**
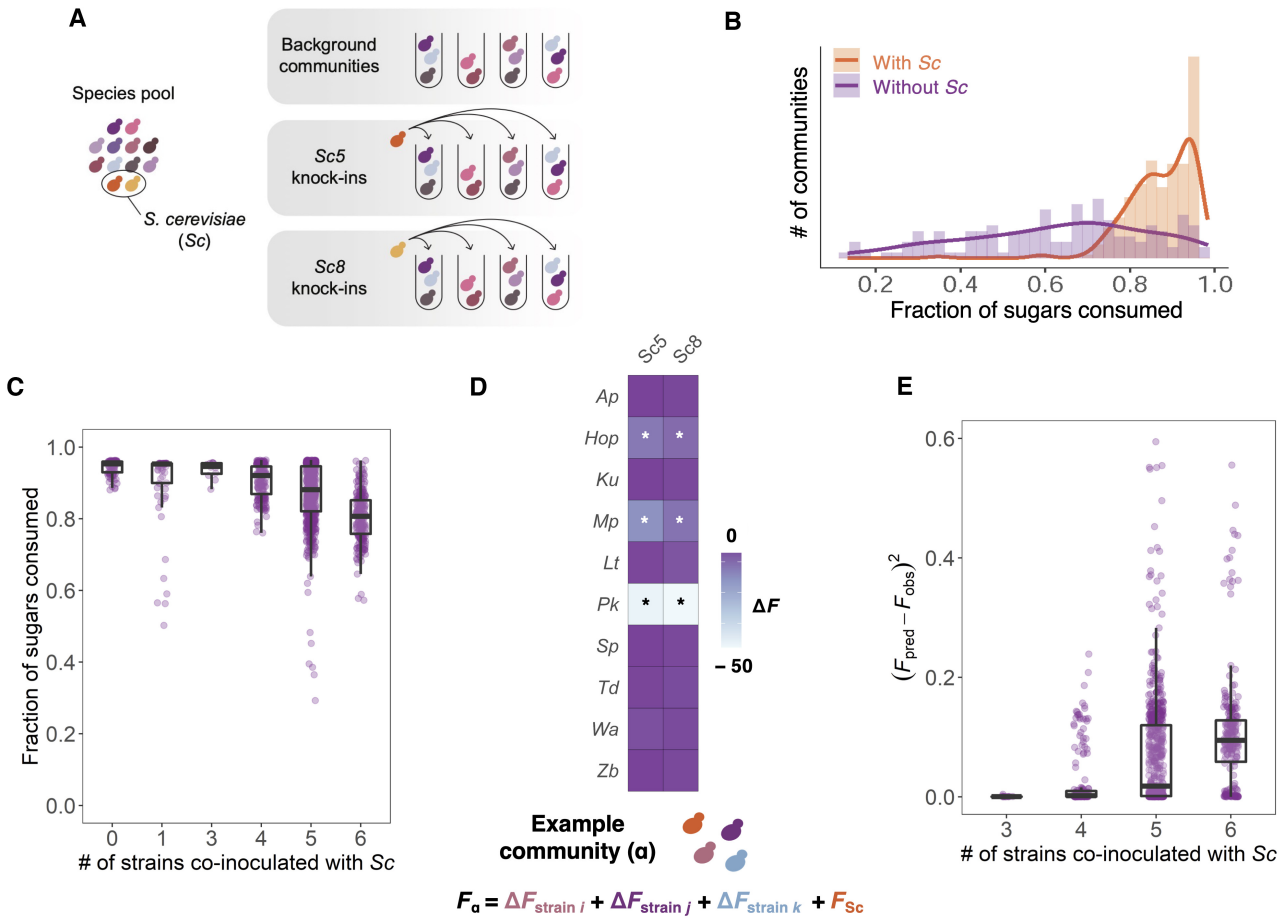
Ecological interactions affect the function and predictability of wine yeast communities To better understand how the complexity of a wine yeast community affects the fermentation performance of *S. cerevisiae*, we constructed synthetic communities of growing diversity. Based on the effect of the different species on the growth efficiency of co‐cultures with *S. cerevisiae* (Appendix Fig S9), we have selected 10 yeast strains (Hop_1, Ku_1, Mp_3, Pk_3, Wa_5, Ap_1, Lt_2, Sp_2, Td_8, Zb_1) to randomly assemble 176 communities (from 2 to 6 members)—named as background communities. These background communities were assayed by themselves and co‐inoculated, in separate experiments, with two different *S. cerevisiae* strains (Sc_5 and Sc_8), constituting a total of 528 synthetic communities assayed. In addition, we assayed the function of all the 12 yeasts studied (10 non‐*Saccharomyces* and the two *S. cerevisiae* strains) individually and in pairwise combinations. Thus, we performed a total of 606 assays, with three replicates, and after 168 h of fermentation in SGM, we measured the fraction of sugars consumed (raw data on sugar consumption of each assay is available in Dataset EV3). A schematic representation of the methodology followed to construct the communities can be found in the Materials and Methods section and in Appendix Fig S17.Despite the ability of *S. cerevisiae* strains to complete wine fermentation (depleting most of the sugars), we observe that certain background communities prevent *S. cerevisiae* to complete the consumption of fermentable sugars.We find that, in our experimental conditions (since we preselected yeast strains based on their effect on *S. cerevisiae* growth efficiency), increasing the richness of the background community increases our chances to detect negative interactions in the community. Each dot represents the fraction of sugars consumed by each community in which *S. cerevisiae* strains were inoculated. We discard a direct effect of richness on community function, since Appendix Fig S13 shows no effect in the function of communities of growing richness, when they only contain the five strains that were preselected with a neutral or positive effect on the growth efficiency of co‐cultures with *S. cerevisiae*.Based on the individual functional effect (ΔF) of the strains on *S. cerevisiae* sugar consumption capacity, we established an additive model where the expected function of a given community was calculated by adding the decreasing effect on function caused by every strain included in that community. Asterisks indicate the existence of statistically significant differences between the functional effect (ΔF) of strains in co‐culture of *S. cerevisiae* compared with *S. cerevisiae* single culture (ANOVA, *P* < 2e‐16).The prediction accuracy of this model (calculated as the squared absolute difference between the observed and the predicted function value of the function) decreases as the diversity of the communities increases, failing to predict the ecological function of complex communities (root‐mean‐square error of each community complexity is: *RMSE*_*3*_ = 0.026; *RMSE*_*4*_ = 0.159; *RMSE*_*5*_ = 0.262; *RMSE*_*6*_ = 0.323). Data information: Boxplot central bands indicate median; box limits indicate 25^th^ to 75^th^ percentiles; and whiskers indicate 1.5 × interquartile range. Each dot represents the function value (panel C)/the prediction accuracy (panel E) of the communities containing *S. cerevisiae* under different levels of strain richness (number of strains co‐inoculates with *Sc*: 0 (*n* = 6 replicates), 1 (*n* = 73 replicates), 3 (*n* = 12 replicates), 4 (*n* = 189 replicates), 5 (*n* = 624 replicates) and 6 (*n* = 231 replicates)). Source data are available online for this figure.

The number of potential background communities one may form out of our 60‐strain library exceeds 10^18^. To be able to tackle this problem empirically, we must therefore first reduce the dimensionality of the problem. To do this in an unbiased manner, we selected a subset of 10 non‐*Saccharomyces* strains from our collection based on their effect on the growth efficiency in co‐cultures with *S. cerevisiae* (Appendix Fig S9), including five strains that lower the growth efficiency of co‐cultures with *S. cerevisiae* (*Hanseniaspora opuntiae* Hop_1 (Hop), *Kazachstania unispora* Ku_1 (Ku), *Metschnikowia pulcherrima* Mp_3 (Mp), *Pichia kudriavzevii* Pk_3 (Pk), and *Wickerhamomyces anomalus* Wa_4 (Wa)); and five strains that do not reduce the growth efficiency of co‐cultures with *S. cerevisiae* (*Aureobasidium pullulans* Ap_1 (Ap), *Lachancea thermotolerans* Lt_2 (Lt), *Schizosaccharomyces pombe* Sp_2 (Sp), *Torulaspora delbrueckii* Td_8 (Td) and *Zygosaccharomyces bailii* Zb_1 (Zb)). Then, we assembled a total of 176 background communities by random combinations of these 10 strains in communities of 2–6 species (with the only constraint of ensuring a similar prevalence of all the species in the set of communities assembled). We assayed the function (final fraction of sugars consumed) of these 176 background communities, both by themselves and by adding, in separate experiments, two *S. cerevisiae* wine strains (Sc_5 and Sc_8; Fig 3A; Appendix Fig S17).

As expected, communities containing *S. cerevisiae* consumed, on average, a higher fraction of sugars than the respective background communities which contained only non‐*Saccharomyces* strains. However, some communities containing *S. cerevisiae* fell far from completing the fermentation, leaving, in some cases, ~30% of residual sugars unconsumed (Fig 3B). We observe that, in our experimental conditions, increasing the richness of the background community increases the chances to detect negative interactions in the community, preventing *S. cerevisiae* to complete the sugar consumption (Fig 3C; Appendix Fig S10). Thus, we confirmed that the diversity of the background community where *S. cerevisiae* is added plays an important role in wine fermentation performance.

A key question is then to what extent interactions among community members determine the quantitative function of these communities, or if, alternatively, knowing the individual effect of yeast strains on the sugar consumption capacity of *S. cerevisiae* can be enough to predict the function of complex communities. We observed that the presence of strains that caused a negative effect on *S. cerevisiae* function, that is Hop, Mp and Pk (Fig 3D) can also anticipate a loss of function in complex communities, since they are more frequently found in communities that were not able to complete the consumption of sugars in SGM fermentations (Appendix Figs S10–S12). Thus, knowing the individual effect of yeast strains can be useful to qualitatively infer their effect on the function of complex communities. However, by exploring an additive model—where the community function is calculated by the sum of the individual effect of each strain in the sugar consumption of co‐cultures with *S. cerevisiae* (Fig 3D)—we are far from quantitatively predicting the function of complex communities (Fig 3E). This suggests the existence of a complex network of interactions—beyond the individual interactions of yeast strains with *S. cerevisiae*—that ultimately shapes the function of wine yeast communities and that should be considered to accurately predict their function.

**Figure 4:**
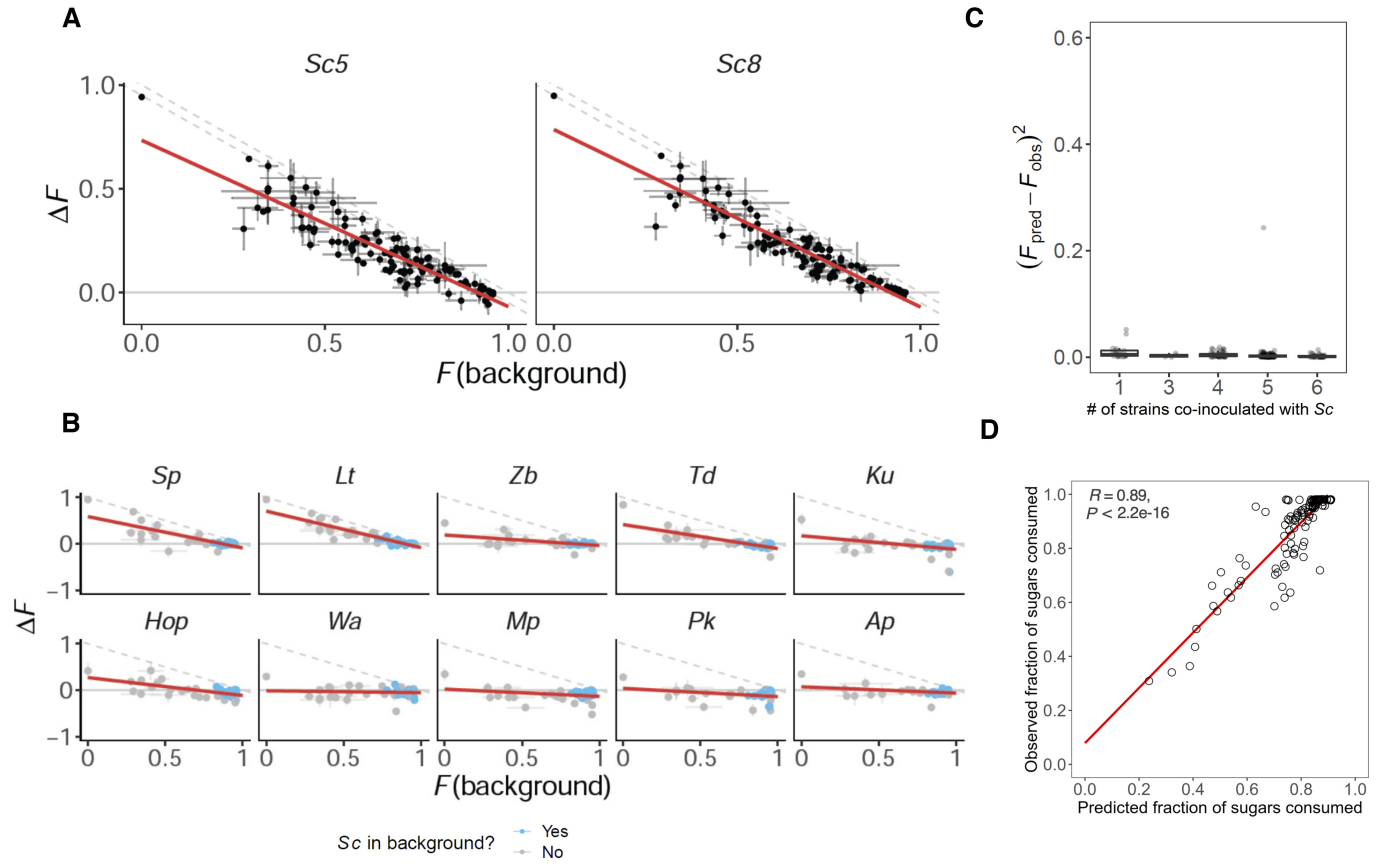
By mapping the ecological interaction occurring within complex wine yeast communities, we can quantitatively predict their function The contribution of the different yeast strains to wine ecosystem function depends on both their own sugar consumption capacity and the function of the background community to which it is added. We show the functional effect (ΔF) of the two *S. cerevisiae* strains (Sc_5 and Sc_8) by representing the change in community function caused by the inoculation of *S. cerevisiae* strain in various backgrounds. The functional effect is defined as the difference in the fraction of sugars consumed by the community with *S. cerevisiae* and the fraction of sugars consumed by the same community without the *S. cerevisiae* strain (F(background)). Dots and error bars represent means and standard deviations of the three biological replicates. Dashed lines represent the functional effect of the strains if its inoculation would cause the consumption of 100% of the sugar (described as *y = 1* − *x*) and the consumption of 95% of the sugars, representing the theoretical and empirical situation of *S. cerevisiae* function when it is inoculated in monoculture. We refer to the linear fits as the FEEs.FEEs of the 10 non‐*Saccharomyces* strains assayed in this experiment. Consistent with similar findings in a wide range of other ecosystems (preprint: Diaz‐Colunga *et al*, 2023), each of the non‐*Saccharomyces* strains has its own FEE. Blue dots represent the background communities that do contain *S. cerevisiae*. Thus, we can study the effect on *S. cerevisiae* fermentation performance on its ecological context (Appendix Fig S14) and predict the function (final fraction of sugars consumed) of this community by sequentially adding the functional effects of each strain that composes each community.This method allows us to accurately predict the function of the community regardless of its complexity. Boxplot central bands indicate median; box limits indicate 25^th^ to 75^th^ percentiles; and whiskers indicate 1.5 × interquartile range. Each dot represents the prediction accuracy of the communities containing *S. cerevisiae* under different levels of strain richness (number of strains co‐inoculates with *Sc*: 1 (*n* = 73 replicates), 3 (*n* = 12 replicates), 4 (*n* = 189 replicates), 5 (*n* = 624 replicates) and 6 (*n* = 231 replicates)).In order to test the prediction accuracy of this FEEs‐based model to predict the function of any randomly assembled community, we assembled 131 new communities (never assayed before) and measured the fraction of sugars consumed after the fermentation. We compared the predicted function of these communities with the measured function, finding a high correlation between the observation and the prediction (*R* = 0.89; Table EV6). Source data are available online for this figure.

However, the number of these possible interactions rapidly explodes with community size, and it is not obvious how one should integrate them even if we knew them. A recent work (preprint: Diaz‐Colunga *et al*, 2023) has found that the collective effect of these interactions is the emergence of simple linear models that predict how each species affects ecosystem function when placed in different community contexts. Moreover, the function of a complex microbial community can be predicted by integrating these linear models. These linear models relate the change in ecosystem function caused by the inoculation of a new focal strain into a new background community (*functional effect*, ΔF) and the function of a background community (background community function, F). We sought to apply this theoretical framework to address the challenge of quantitatively predicting the functional effect of different wine yeast strains in the presence of interactions. Our experimental approach allowed us to compare the fraction of sugars consumed by a background community with the change in that fraction produced by both the two *S. cerevisiae* strains (Fig 4A) and by all the 10 non‐*Saccharomyces* strains assayed (Fig 4B). In this way, we can characterise the ecological effect of these strains by analysing the slope and intercept of the linear models that predict their functional effects in different community backgrounds (these models are referred to as functional effect equations, or FEEs). As expected, we observe high intercepts for both fits of *S. cerevisiae* strains, since they showed the highest individual function (they were able to mostly deplete 95% of sugars as a single inoculum). However, note that if *S. cerevisiae* were able to consume 100% of the sugars (or, at least, 95%, according to what we have empirically observed in our experimental condition when *S. cerevisiae* is inoculated in monoculture) regardless of the composition of its accompanying community, its functional effect equation would be well described by a line of the form *y* = 1 − *x* (dashed lines in Fig 4A). Instead, we found that the functional effect equations of both *Sc_5* and *Sc_8* fall below this line, reflecting that *S. cerevisiae* strains are unable to complete wine fermentations in some ecological contexts.

Likewise, Fig 4B shows that the functional effects of the 10 non‐*Saccharomyces* strains assayed are also well described by linear fits. The high fermentative yeasts *S. pombe* (Sp), *L. thermotolerans* (Lt) and *T. delbrueckii* (Td) exhibit FEEs that are very similar to that of the two *S. cerevisiae* strains. However, some strains (mainly Mp and Pk, but also Wa and Ap) exhibited a marked negative effect on the function of the community, and the function of the background community has little to no effect in magnitude of their impact. Other strains, such as Hop—or more subtly Ku*—*have functional effects that are positive in low‐functioning backgrounds but negative in high‐functioning backgrounds (i.e. communities with *S. cerevisiae* strains; Appendix Figs S11 and S12). Therefore, this approach was useful to more accurately characterise the qualitative contribution of each strain in different ecological contexts, allowing us to estimate if they might facilitate or prevent *S. cerevisiae* from completing fermentation. But further, here we demonstrate that by concatenating the FEEs of each strain member of the community (preprint: Diaz‐Colunga *et al*, 2023), we can quantitatively predict the function of any randomly assembled wine yeast community, independently of their diversity level (Fig 4C). To double‐check the robustness and accuracy of this model, we assembled and assayed 131 new yeasts consortia that have not been assayed in the first experiment used to fit the model, comparing the predicted and observed values for their sugar consumption capacity (Table EV6). This assay was conducted under identical conditions than the first experiment, but it was carried out by a different member of the laboratory. As shown in Fig 4D, we found a significant strong correlation between predicted and observed functions (*R* = 0.89). We confirm that knowing the individual behaviour of each species in different ecological contexts, we can quantitatively link the taxa composition of complex communities to the ultimate function they provide.

Additionally, given the success we had in using phylogenetic imputation to predict ecologically relevant traits in wine yeasts (Appendix Fig S5), we asked whether the FEE slope and intercept could also be interpreted as complex ecological traits and, thus, can be predicted in the same manner. Appendix Fig S15A shows how FEEs parameters are clustered based on the phylogenetic relationships among the strains. We can observe a marginally significant correlation between observed and predicted FEEs parameters; *R* = 0.45 for slope (Appendix Fig S15B) and *R* = 0.47 for intercept (Appendix Fig S15C), but it seems that the functional effect of yeast strains in complex communities cannot be clearly predicted by phylogeny. The more limited size (*n* = 12) of our strains collection assayed in complex consortia, and the strong impact of the function of the background communities might explain this lack of predictability.

Moreover, we also looked for individual traits (among those described in Fig 2) that can be somehow related to the ecological effect of strains (slope and intercept in the FEEs) in wine yeast communities. Although further work will be necessary to further establish causal links between the metabolic traits of a given species and its contribution to the ecosystem function, we found that the ethanol production capacity, the consumption of organic nitrogen and the glucose:fructose consumption ratio are important predictors of the effect that one strain may have on the function of wine yeast communities (Appendix Fig S16C and D).

In particular, we will address future works to elucidate the molecular and ecological mechanisms of action of those yeasts found here as leading to stuck wine fermentations (mainly *P. kudriavzevii*, *H. opuntiae* and *M. pulcherrima*), but, for now, here we just provide evidences that, even in microbial communities usually dominated by a single highly adapted species, interspecies interactions play a crucial role in ecosystem function.

## Discussion

Using wine fermentations as a model ecosystem, we have focussed on a major question in microbial ecology, studying how strain‐level traits and taxonomic composition shape the ecological function of complex communities. We first show that key functional traits of ecological and industrial significance exhibit phylogenetic signal. The phylogenetic conservatism of microbial traits is required if we wish to quantitatively predict different ecological functions in a community from its taxonomic composition. However, this cannot be taken for granted (Martiny *et al*, 2013): the genetic and physiological plasticity of microorganisms and their rapid evolutionary adaptation rates could suppose a weaker phylogenetic signal in particular traits or systems (Blomberg *et al*, 2003; Hertz *et al*, 2013; Krause *et al*, 2014).

Our results indicate a significant correlation between the phylogenetic and phenotypic distance of wine yeast species, but it is much stronger on the environmental preferences traits (Fig 2C and Appendix Fig S3). Due to the inherent experimental nature of determining the fermentative performance traits, there may be several physicochemical variables during fermentation that can interact with the final concentration of the specific metabolites measured, resulting in the weaker correlation observed. Despite these limitations, the correlation observed allowed the detection of a significant phylogenetic signal for all traits analysed (Fig 2D). Martiny *et al* (2013) discussed that complex traits are more phylogenetically conserved than simpler traits that involve one or few genes, such as the consumption of a specific nutrient. In our work, most traits studied are based on the ability of yeasts to grow in synthetic grape must, that is fermentation capacity. This capacity is associated with some specific events in the evolutionary history of yeast (i.e. whole‐genome duplication and the loss of the Respiratory Complex I; Dashko *et al*, 2014), providing a plausible explanation for why this trait is strongly conserved in the phylogeny. Interestingly, the only trait analysed that did not show a significant phylogenetic signal is related to malic acid consumption capacity of yeasts. The use of malic acid as carbon source requires the yeast to import and metabolise L‐malate, using a malate transporter (*S. cerevisiae* lacks an active transport system for L‐malate), and a decarboxylating malate dehydrogenase (the malic enzyme), that can be mitochondrial (less active) or cytosolic. More studies will be necessary to explore the evolutionary origin of malic acid consumption capacity in phylogenetically distant wine yeast species (Saayman & Viljoen‐Bloom, 2006).

Furthermore, we highlight the usefulness of phylogeny‐based imputation (Goberna & Verdú, 2016) as a valuable tool for yeast researchers. The observed phylogenetic signal enables us to predict the environmental preferences of a wide range of yeast species, and to a lesser but still significant extent, their enological properties. During the last decade, high‐throughput sequencing studies in wine fermentations revealed a greater‐than‐expected yeast diversity in wine fermentations (Bokulich *et al*, 2016; Liu *et al*, 2017; de Celis *et al*, 2022), increasing the number of potentially relevant species to be characterised. Thus, we stand out the relevance of the dataset and predictive tools provided in this work, which can help to easily predict wine‐relevant traits of new taxa, only requiring their phylogenetic information provided by a single gene marker (26S sequence). Although a single gene marker has limited genomic resolution and—in some cases is insufficient to distinguish between two strains of the same species (as shown in Appendix Figs S1 and S7)—the phylogenetic imputation applied in this work is effective enough to overcome these limitations, despite the high intraspecific diversity found in our collection. In fact, we have conducted the LOOCV method at various taxonomic levels to assess the robustness of the method (Appendix Fig S6). We observe decreasing correlations between predicted and observed trait values when we repeat the imputation leaving out the closest relatives with identical 26S sequences (Appendix Fig S6B), compared with the imputation using all the strains of the collection (Appendix Fig S6A). As expected, the correlation between predicted and observed trait values decreases as closely related strains are removed. Nevertheless, even when trait values are imputed using information at the species level, we find that 29/43 traits show a significant positive correlation between predicted and observed trait values (Appendix Fig S6C). And yet, even in the most unfavourable situation—where phenotypes are imputed without using information from any strains in the same genus—we observe a significant positive correlation in 22/43 traits (Appendix Fig S6D). These results suggest that despite the immense complexity of genotype–phenotype relationships, a single marker gene may still be useful for predicting ecologically relevant quantitative traits when placed in a broader phylogenetic context.

Several previous works have explored in detail the intraspecific phenotypic diversity of some relevant wine yeasts, such as *S. cerevisiae* (Camarasa *et al*, 2011), *L. thermotolerans* (Hranilovic *et al*, 2018) or *T. delbrueckii* (Silva‐Sousa *et al*, 2022). Here, looking at the interspecies diversity, we provide information about ecologically and industrially relevant traits of 60 wine strains belonging to 30 different yeast species. We hope that our yeast collection and its phylo‐functional characterisation would also be a useful resource for wine researchers to guide future wine yeast selection programmes. In this regard, we highlight some outstanding results, which may help, for instance, to contribute to better manage the effects of climate change in the wine industry, such as the capacity of *T. delbrueckii* to grow in vitamins‐deficient grape musts, or to modulate wine acidity by producing lactic acid (*L. elongisporus* and *L. thermotolerans*) or degrading malic (*H. osmophila*, *L. elongisporus* and *P. kudriavzevii*) or tartaric acid (*C. amylolentus*; Fig 2A and B).

Once we have established that there exists a strong enough link between taxonomy and function within the wine yeast microbiome, we sought to understand the effect of interspecies interaction patterns in modulating the functional contributions of each community member and in shaping the ecological function of complex wine yeast communities. *S. cerevisiae* becomes the dominant yeast during wine fermentations, as it can rapidly complete wine fermentations by consuming almost all fermentable sugars from the grape must by its own. This ability is result from the adaptation of *S. cerevisiae* to the challenging conditions of wine fermentations (Marsit & Dequin, 2015; García‐Ríos & Guillamón, 2022), due to several genomic features that wine strains acquired over their domestication process (Belda *et al*, 2019). However, we observed that some combinations of yeast strains caused a negative effect on *S. cerevisiae* function (Fig 4B; Appendix Figs S10 and S11). This suggests that the accompanying yeast species—despite their limited sugar consumption capacity—can potentially prevent *S. cerevisiae* from completing wine fermentation. The results depicted in Appendix Fig S16 have prompted us to anticipate certain specific traits, such as ethanol production and nitrogen source consumption, that may elucidate the negative impact of some species shown under our experimental conditions, defined by the FEEs parameters intercept (Appendix Fig S16A) and slope (Appendix Fig S16B). In addition, some interaction phenomena against *S. cerevisiae* have been already described, such as the production of inhibitory pulcherriminic acid by *Metschnikowia* yeasts (Gore‐Lloyd *et al*, 2019) or the high nitrogen consumption capacity of *K. unispora* (Vicente *et al*, 2023). Yet, little is known about these effects under complex community contexts, where, as we demonstrated here, higher‐order interactions emerge. Although this study aims to identify and quantify the effects of these interactions rather than elucidating the underlying mechanisms, we hope these results provide valuable insights for designing future mechanistic studies aimed to unravelling the molecular and physiological basis of wine yeast interspecific interaction.

The effect of these species on the community function cannot be accurately explained by the additive effect of the individual interaction patterns of each accompanying species with *S. cerevisiae* (Fig 4E). This indicates the importance of interactions in determining the effect of complex microbial communities on *S. cerevisiae* fermentation performance. Supporting this idea, a recent work of Conacher *et al* (2022) has evidenced the presence of high‐order interactions in wine yeast consortia, demonstrating that the inoculation of *S. cerevisiae* with complex yeast communities disclose transcriptional responses not explained by the transcriptional responses observed in pairwise co‐culture.

If we aspire to engineer microbiomes for biotechnological purposes, we need to be able to predict the function of complex communities, guiding the search for the best possible combination of species. In principle, quantitatively predicting the fraction of sugars consumed by a particular combination of yeast species during wine fermentations, might seem like a challenging task, requiring extensive characterisation of the chemical and physiological mechanisms through which yeast strains may interact with one another. However, we demonstrated that the contribution of different wine yeast species to the function of complex communities can be described by linear patterns based on their own sugar consumption capacity in different ecological contexts. We show that, by characterising these patterns, we could not only improve our understanding of the ecology of wine yeasts (Fig 4A and B, and Appendix Fig S14), but also use this information (FEEs) to fit a model that allows us to quantitatively predict the ecological function of the wine yeast communities. Herein, we have taken advantage of the theoretical framework established by preprint: Diaz‐Colunga *et al* (2023) to improve the understanding of a highly valuable biotechnological process such as wine fermentation.

By investigating the microbial diversity and the interactions that take place within it, we can gain a better understanding of microbial fermentations. This knowledge can contribute to the design of starter cultures and the development of scientifically guided fermentation processes (Jahn *et al*, 2023). To conclude, with this work we want to encourage the use of fermented foods as model systems (Wolfe & Dutton, 2015) to address fundamental questions in ecology and evolution but also to adopt the conceptual frameworks of these disciplines for a more integrated understanding of microbial‐based industrial processes.

## Materials and Methods

### Yeast strains and molecular identification

Yeast strains used in this study are listed in Dataset EV1 (main collection of 60 yeast strains used in the study) and Table EV3 (list of 53 additional yeast strains used for double‐checking phylogeny‐based phenotypic predictions (Appendix Fig S7)). All yeast isolates were identified by sequencing the D1/D2 domain of the 26S large subunit of rRNA gene, with the forward NL‐1 primer (5′‐GCATATCAATAAGCGGAGGAAAAG‐3′) and the reverse NL‐4 (5′‐GGTCCGTGTTTCAAGACGG‐3′) primer. After Sanger sequencing, the sequences obtained were compared and identified by BLAST‐search and submitted to the GenBank database (NCBI accession numbers are detailed in Dataset EV1 and Table EV3). *Sabouraud* Dextrose Agar medium (glucose 20 g/l; peptone 20 g/l; yeast extract 10 g/l and 15 g/l agar) was routinely used for handling the strains.

### Estimation of population prevalence and relative abundance of yeast genera in wine samples

The population prevalence and relative abundance patterns of the yeast genera represented in our yeast collection (showed in Appendix Fig S2) were calculated using the dataset reported in de Celis *et al* (2022), where we analysed a total of 144 wine fermentation samples following an ITS‐amplicon sequencing strategy. Raw sequences (fastQ files) are available at NCBI (Bioproject: PRJNA814622); and details about DNA extraction, sequencing, bioinformatics and taxonomic assignment are described in the work by de Celis *et al* (2022). In the particular case of the genus *Metschnikowia*, its population prevalence and relative abundance figures were obtained from the work of Vicente *et al* (2020).

### Phylogenetic tree reconstruction

Sequences from the D1/D2 domain of the 26S large subunit of rRNA gene of the 60 yeast strains (NCBI accession codes detailed in Dataset EV1) were used to construct a phylogenetic tree (Appendix Fig S1). We aligned the 113 sequences using the *msa* package (v3.16; Bodenhofer *et al*, 2015) in R (v 4.2.2), implementing the ClustalW algorithm. The alignment was trimmed using trimAl (automated1) to remove poorly aligned regions (Capella‐Gutiérrez *et al*, 2009). After trimming, we inferred a maximum likelihood phylogenetic tree using IQTREE 2 with 1,000 bootstrap replicates (‘iqtree2 ‐s alignment.phy ‐bb 1,000 ‐redo’). The consensus tree was rooted using the Basidiomycota clade as an outgroup as this represents the deepest known taxonomic division among all yeast strains. An ultrametric tree was then obtained using the chronos function in the *ape* R package (v5.6‐2), and all subsequent analysis was performed on this tree. Heatmaps showing trait distributions across the phylogenies (Fig 2A and B) were constructed using the *ggtree* R package (v3.16; Yu, 2020).

### Study of environmental preferences (growth assay) of the yeast collection

To study the environmental preferences of our yeast collection (represented in Fig 2A), the 60 yeast strains were assayed in a panel of 28 growing conditions of enological interest (detailed information about the composition of culture media and growing conditions is available in Table EV1), in brief: 16 wine fermentation conditions (growth assays in synthetic grape must‐based media), the capacity to grow using the most relevant organic acids of grape musts as sole carbon sources (malic acid, citric acid and tartaric acid; plus glucose as a control), the most abundant amino acids of grape musts as sole nitrogen sources (lysine, serine, threonine, arginine, glutamic acid and proline; plus ammonia as a control), and a standard medium (Yeast Nitrogen Base (YNB, BD Difco™, USA)) without amino acids and (NH₄)₂SO₄ (1.7 g/l); glucose (10 g/l); as a growth control condition.

Synthetic grape must (SGM) was prepared as described by Henschke & Jiranek (1993), with the modifications detailed below. First, vitamins (20×), amino acids (20×), trace elements (1,000×) and anaerobic factor (200×; ergosterol, Tween 80 and ethanol) stock solutions were prepared separately, sterilised by 0.45 μm filtration and stored at 4°C (maintaining the proportion of the original recipe). To prepare the SGM, the stock solutions (50 ml of vitamins; 10.79 ml of amino acids stock; 1 ml of trace elements stock and 5 ml of anaerobic factor stock) were dissolved in H_2_O followed by glucose (100 g/l), fructose (100 g/l), malic acid (3 g/l), potassium L‐tartrate (2.5 g/l), citric acid (0.2 g/l), KH_2_PO_4_ (1.14 g/l), MgSO_4_·7H_2_0 (1.23 g/l), CaCl_2_·2H_2_O (0.44 g/l) and (NH_4_)_2_HPO_4_ (0.24 g/l). Then, pH was set to 3.5 with KOH and SGM was sterilised by 0.45 μm filtration. Yeast assimilable nitrogen (YAN) was adjusted to 200 mg N/L (60 mg N/L of ammonia‐nitrogen ((NH_4_)_2_HPO_4_) and 140 mg N/L of amino acids‐nitrogen). This SGM medium was used for assaying the growth capacity of yeasts in 16 wine fermentation conditions, with the required modifications in each case (Table EV1). The growth capacity using different carbon sources was assayed in Synthetic Medium (SM) prepared with YNB (1.7 g/l) and 1 g/l of the corresponding carbon source (malic acid, citric acid, tartaric acid and glucose as a control). Likewise, the growth capacity using different nitrogen sources was assayed in SM prepared with Yeast Carbon Base (1.7 g/l; YCB, BD Difco™, USA), and 200 mg N/L of the corresponding nitrogen source (serine, proline, lysine, arginine, threonine, glutamine and ammonia as a control). All media were sterilised by 0.45 μm filtration.

To perform these assays, strains were streaked out on *Sabouraud* Dextrose Agar medium plates and grown at 28°C during 24 h. Then, strains were precultivated for 24 h on 250 μl of SGM in a 96‐well microplate. After that, the strains were inoculated, as biological triplicates, at a final optical density (OD_600nm_) of 0.01, in 96‐well plates filled with 250 μl of the corresponding medium. Assays were performed at 25°C (except the fermentation assays at low (15°C) and high (32°C) temperatures) under orbital shaking set at 100 rpm. Yeast growth was monitored by measuring OD_600nm_ at different time points for 72 h, using the microplate reader Varioskan Flash Multimode Reader (Thermo Scientific, USA). Growth efficiency (total variation in cellular density) was extracted by using *GrowthRates* package (v0.8.4; Hall *et al*, 2014) in R, adjusting the growth curves to a Baranyi model.

### Synthetic grape must microvinification assays

The fermentation performance of the 60 yeast strains of our collection (that is: their contribution to the chemical profile of wine, determined by measuring 15 physicochemical parameters at the end of fermentations) was studied in microvinification assays using SGM (results presented in Fig 2B). First, strains were precultured during 24 h in 12‐well plates containing 3 ml of SGM at 25°C under orbital shaking (100 rpm). Then, strains were inoculated in SGM at a final OD_600nm_ of 0.01. All fermentations were performed as biological triplicates at 25°C (under orbital shaking at 100 rpm) in 15 ml plastic tubes filled with 14 ml of SGM. After 168 h of fermentation, cultures were centrifuged at 4,500 x *g* for 10 min to remove biomass. Then, supernatants were stored at −20°C until further analysis to determine their fermentation performance (Table EV2; Dataset EV2). Wine pH, total acidity, volatile acidity and the concentration of ethanol, malic acid, lactic acid, glucose and fructose were determined by Fourier‐transform infrared spectroscopy (FTIR), using an infrared analyser (Bachus 3 MultiSpec, *Tecnología Difusión Ibérica*, S.L, Spain). The concentration of ammonia and primary amino nitrogen (PAN) was determined enzymatically by using the appropriate kits (Biosystems, Barcelona, Spain) and a spectrophotometric autoanalyzer Y15 (Biosystems).

### Phylogenetic signal analysis

The phylogenetic (cophenetic) distance between yeast strains was calculated as the sum of branch lengths between pairs in the maximum likelihood tree (Appendix Fig S1). Phenotypic distance between yeast strains was calculated as the Euclidean distances on the scaled trait values, both from the environmental preferences and the fermentation performance of the studied strains (Dataset EV2; shown in Fig 2A and B). For each trait, we quantified the phylogenetic signal (Pagel's λ) by using the *phylosig* function from the *phytools* R package (v0.1‐9; Revel, 2012). Pagel's λ parameter represents a continuous measure of phylogenetic signal that varies from 0 to 1. λ = 0 indicates that the trait has evolved independently of the phylogeny whereas λ = 1 indicates that the trait has evolved according to a Brownian motion model of evolution. The statistical significance of λ was tested using a permutation test where trait values were randomly permuted across the tree and λ_perm_ was recalculated for each permutation (Münkemüller *et al*, 2012). λ was determined to be statistically significant if λ > λ_perm_ in more than 95% of permutation.

### Predicting wine yeast traits with phylogeny

For each of the 43 phenotypic traits measured (28 growing conditions and 15 physicochemical parameters), we tested whether it is possible to predict the traits of a novel strain in the phylogeny using the phylogenetic imputation algorithm that assumes a Brownian motion model of evolution. To do that we used the *phylopars* R package (Goolsby *et al*, 2017). Predictions were performed using the Leave One Out Cross‐Validation (LOOCV) method. Specifically, we removed all trait values associated with a given strain before fitting and imputed the missing value using the phylopars algorithm. Observed and predicted trait values were then compared. The predictive accuracy of the algorithm was quantified for each trait by calculating the Pearsons' correlation coefficient between predicted and observed values. A wider collection of yeast strains (the 60 strains already characterised, plus 53 new strains; Table EV3) was used to check the accuracy of phylogenetically based predictions. The growth efficiency parameter in SGM was measured for the 113 strains (Table EV4). Thus, 60 strains have been assayed in two independent batches of experiments, and 53 strains have been assayed once. We used this information to compare the prediction based on the phylogenetic imputation method trained with the original dataset of 60 strains and the accuracy of the replicate measurements of the same strain in independent experiments. This phylogenetic imputation algorithm was also used to explore the predictability of the main FEEs parameters (slope and intercept) based on phylogeny, as shown in Appendix Fig S15.

### Co‐culture assays of *S. cerevisiae* and non‐*Saccharomyces* strains

We measured the effect of the non‐*Saccharomyces* strains on the growth efficiency of co‐cultures with *S. cerevisiae* (results shown in Appendix Fig S9; Table EV5). Co‐culture growth assays were performed by co‐inoculating the 60 strains of the study with *S. cerevisiae* Sc_5 strain (1:1 proportion, at a final OD_600nm_ of 0.02) in SGM media. Sc_5 × Sc_5 co‐culture was also assayed, as a control, at a final OD_600nm_ of 0.02. These assays were performed, in six biological replicates, as described previously for the environmental preferences growth phenotyping (in 96‐well plates filled with 250 μl of SGM, at 25°C with 100 rpm agitation, for 72 h), and the growth efficiency (total variation in cells density) values were obtained from growth curves as previously described. The effect of each strain on the co‐culture function (growth efficiency) was expressed as Δ*F* = *F*_*i* + Sc_ − *F*_Sc_, where *F*_*i* + Sc_ is the function of the co‐culture when the strain *i* is co‐inoculated with *S. cerevisiae* strain, and *F*_Sc_ is the function of *S. cerevisiae* single culture (Sc_5 × Sc_5 assay). This experiment allowed us to identify five strains that caused an increase on the co‐culture function (Ap_1, Lt_2, Sp_2, Td_8, Zb_1) and five strains that caused a decrease on the co‐culture function (Hop_2, Ku_1, Mp_3, Pk_3, Wa_4) compared with *S. cerevisiae* single culture. These strains were selected for the subsequent construction of synthetic yeast consortia, as explained below.

### Construction and study of synthetic consortia of wine yeasts

To test how the composition of a background community can affect the ecological function of *S. cerevisiae* in wine fermentation, we assembled a total of 176 synthetic consortia combining the 10 non‐*Saccharomyces* strains mentioned before (*Ap_1*, *Lt_2*, *Sp_2*, *Td_8*, *Zb_1*, *Hop_2*, *Ku_1*, *Mp_3*, *Pk_3*, *Wa_4*). The assembly of the synthetic consortia was carried out as represented in Appendix Fig S17: first, we designed 16 background communities (between 3 and 6 species) by random combinations of the 10 non‐*Saccharomyces* strains (with the only constraint of ensuring a similar prevalence of all the species in the set of communities assembled), and then, we also added each of the 10 species on each background community, to determine their functional effect in different ecological contexts (the composition and function of all synthetic yeast consortia assayed can be found in Dataset EV3). These 176 communities were then assayed by measuring their sugar consumption capacity in SGM (explained in the following section), both by themselves and by adding, in separate experiments, two *S. cerevisiae* wine strains (Sc_5 and Sc_8), resulting in a total of 528 combinations assayed as biological triplicates. All yeast strains (10 non‐*Saccharomyces* and 2 *S. cerevisiae*) were also assayed individually and in pairwise combinations.

First, yeast strains were streaked out on *Sabouraud* Dextrose Agar medium plates and grown at 28°C during 24 h. After that, single colonies were used to inoculate precultures into 3 ml SGM medium on 12‐well plates. After 24 h of growth at 25°C under orbital shaking (100 rpm), yeast cultures were combined, according to the community design, in 2 ml tubes. Then, 10 μl of the prepared mix culture (synthetic yeast consortia) were inoculated into 96‐well plates to reach a final OD_600nm_ of 0.02 in 250 μl of SGM (each non‐*Saccharomyces* strain member of the community was at a final OD_600nm_ of 0.004). Then, when necessary, 10 μl of the adjusted *S. cerevisiae* cultures were also added into the assays, together with the background community, to reach a final OD_600nm_ of 0.001 (i.e. a 1:4 ratio with each non‐*Saccharomyces* strain in the background, to simulate the lower abundance of *S. cerevisiae* in relation to the rest of non‐*Saccharomyces* yeasts found in natural yeast communities of grape musts). OD_600nm_ of the cultures was monitored during 168 h as described previously for the growth phenotyping.

### Analysis of the function of synthetic yeast consortia

Sugar consumption was measured as the main ecological function of the assayed synthetic consortia, by the quantification of total fermentable sugars (glucose + fructose) consumed after 168 h of fermentation (results shown in Figs 3 and 4). We used the 3,5‐dinitrosalicylic acid (DNS) () method (Luyt *et al*, 2021), adapted to 96‐well plates. Briefly, in each well 100 μl of the DNS solution (30 g/l potassium sodium tartrate; 16 g/l NaOH; 10 g/l DNS) were incubated (100°C for 30 min) with 10 μl of each sample and then cooled at 4°C for 30 min, and, finally, absorbance was measured at 540 nm in the microplate reader Varioskan Flash Multimode Reader (Thermo Scientific, USA). The absorbance values were interpolated in a calibration curve prepared using the same protocol. Ten half serial dilutions of standard SGM (200 g/l glucose + fructose) diluted with SGM without sugars by triplicate were used for the calibration curve. Concentration values above 12.5 g/l were out of the linearity range of the calibration curve. Therefore, all the assays exceeding this value were measured again, with a previous dilution of 1/10 or 1/20 as necessary. The fraction of sugars consumed by the assembled communities was calculated by the difference between the initial sugar concentration (200 g/l) and the final residual sugar concentration at the end of the fermentation assays and normalised to 0–1 range (Dataset EV3). The functional effect of adding one strain to a specific background community on the ecological function (fraction of sugars consumed) was expressed as Δ*F*_*i*_ = *F*_*j* + *i*_ − *F*_*j*_; where *F*_*j* + *i*_ is the function of the background community *j* when the strain *i* is added and *F*_*j*_ is the function of the background community *j*.

### Simple additive model for the prediction of the function of complex consortia

To demonstrate the existence of higher‐order interactions in the wine yeasts communities we have assayed, firstly created a simple prediction model feed with the individual—detrimental—effect of each strain in co‐cultures with *S. cerevisiae* (Δ*F*). Thus, as an example, the function of the community A (composed by the strains *i*, *j* and *z* and co‐inoculated with the *S. cerevisiae* strain) was predicted by this model as follows: *F*_A_ = Δ*F*_*i*_ + Δ*F*_*j*_ + Δ*F*_*z*_ + *F*_Sc_. To test the accuracy of predictions based on this model, we calculated the root‐mean‐square error (RMSE) by using the rmse function of the *Metrics* package in R (v 4.2.2), which compares the observed values of the function of the communities and the prediction values using the additive model. High RMSE values indicate a low predictability accuracy of the model. For the individual predictions of each community, we calculated the squared absolute difference between the observed and the predicted function value (represented against strain complexity in Figs 3E and 4C).

### Leveraging functional effect equations to predict community function

By following the methodology described by preprint: Diaz‐Colunga *et al* (2023), the functional effect of each strain (Δ*F*) is represented against the function of the different background communities where they are added *F* (A) (Fig 4A and B). Thus, the change in community function (functional effect, Δ*F*) induced by a particular species can be linked to the function of the background community via a linear model of the form

$$\Delta F_{i}\left(B\right)=a_{i}+b_{i}F\left(B\right)+\theta_{i}\left(B\right)$$

where *ΔFi*(*B*) represents the functional effect of strain *i* in background community *B*, *ai* and *bi* are the intercept and slope of the linear model for species *i*, *F*(*B*) is the function of the background community, and *θi*(*B*) is the residual of the fit corresponding to species *i* in background community *B*. Thus, the intercepts and the slope can define the ecological effect of each strain. At this point, we tried to predict the ecological function (fraction of sugars consumed) of the yeast communities assembled based on their taxonomic composition. Thus, by concatenating the FEEs of each strain that composes the community, we can estimate the function of any randomly assembled community (composed by the 12 strains assayed). This methodology is explained in more detail in preprint: Diaz‐Colunga *et al* (2023); however, a brief explanation can be provided as follows:

In general, we want to predict the function of a target community (denoted **c**_t_) which we have not empirically measured (or which we have measured but are leaving out of the sample for cross‐validation). We start by identifying an in‐sample community that contains less species than **c**_t_, but such that all species in it are also present in **c**_t_ — we denote this “starting” community **c**_0_. For instance, if **c**_t_ contains species *A*, *B*, *C* and *D*, we might choose **c**_0_ to be the community that contains species *A* and *B*. Since we have measured the function of **c**_0_ (which we denote *F*(**c**_0_)), we can first estimate the functional effect of species *C* in **c**_0_ as

(1) $$\Delta F_{C}\left(c_{0}\right)=a_{C}+b_{C}F\left(c_{0}\right)+\theta_{C}\left(c_{0}\right)$$

And thus, we estimate the function of the community resulting from the addition of species *C* to **c**_0_ (which we denote **c**_0 + *C*_) as

(2) $$F\left(c_{0+C}\right)=F\left(c_{0}\right)+\Delta F_{C}\left(c_{0}\right)=F\left(c_{0}\right)+a_{C}+b_{C}F\left(c_{0}\right)+\theta_{C}\left(c_{0}\right)$$

Next, we need to estimate the effect of adding species *D* to the community **c**_0 + *C*_. We can do this by using the functional effect equation for species *D*, simply as

(3) $$\Delta F_{D}\left(c_{0+C}\right)=a_{D}+b_{D}F\left(c_{0+C}\right)+\theta_{D}\left(c_{0+C}\right)$$

Note that, in the expression above, the value of *F*(**c**_0 + *C*_) would just be the estimate provided by equation (2). We can thus estimate the function of the target community **c**_t_ as

(4) $$\begin{array}{rcl} F\left(c_{t}\right) & = & F\left(c_{0}\right)+\Delta F_{C}\left(c_{0}\right)+\Delta F_{D}\left(c_{0+C}\right)\\ & = & F\left(c_{0}\right)+a_{C}+b_{C}F\left(c_{0}\right)+\theta_{C}\left(c_{0}\right)+a_{D}+b_{D}F\left(c_{0+C}\right)+\theta_{D}\left(c_{0+C}\right) \end{array}$$

Note that we can iterate this process as many times as needed if the starting community **c**_0_ and the target community **c**_t_ differ in the presence of more than two species. While the residuals of the fits (θ) are generally unknown, they can be estimated via maximum likelihood as explained in more detail in preprint: Diaz‐Colunga *et al* (2023). Estimating the residuals in this way also ensures that the order in which species are “added” (i.e. the order in which functional effect equations are “concatenated”) does not affect the final predicted value for *F*(**c**_t_).

To double‐check the accuracy of this FEEs‐based prediction model (Fig 4D), 131 new communities—not assembled in the first experiment—were assembled and assayed (Table EV6). Following the experimental procedure described above, the fraction of sugars consumed was calculated after 168 h of SGM fermentation. To also test the replicability of the procedures, this new experiment was conducted by a different lab member. The predicted function values were then compared with the actual measured values to estimate the prediction ability of this model.

### Importance of phenotypic trait as predictors of FEEs slope and intercept

To assess the importance of all the individual phenotypic traits measured (those represented in Fig 2) as predictors of the functional effect of yeast strains on different ecological contexts (i.e. FEEs slope and intercept), we used random forest classification analysis using *randomForest* (v 4.7–1.1 package; Breiman & Cutler, 2018) in R (Appendix Fig S16). We included the environmental preferences and the measured physicochemical parameters (fermentation performance) as predictors of the slope and intercepts of the predicted function (sugar consumption), considered as the response variables. The growth efficiency values in the media used to test the growth ability using different organic acids and amino acids as carbon and nitrogen sources were grouped, respectively, as carbon and nitrogen sources.

## Author contributions

**Javier Ruiz:** Conceptualization; investigation; visualization; methodology; writing – original draft; writing – review and editing. **Miguel de Celis:** Data curation; software; formal analysis; investigation; methodology; writing – original draft. **Juan Diaz‐Colunga:** Data curation; software; formal analysis; visualization; methodology; writing – review and editing. **Jean CC Vila:** Data curation; software; formal analysis; investigation; visualization; methodology; writing – review and editing. **Belen Benitez‐Dominguez:** Validation; investigation; writing – review and editing. **Javier Vicente:** Investigation; methodology; writing – review and editing. **Antonio Santos:** Resources; investigation; writing – review and editing. **Alvaro Sanchez:** Resources; supervision; validation; investigation; writing – original draft; writing – review and editing. **Ignacio Belda:** Conceptualization; resources; supervision; funding acquisition; validation; investigation; visualization; writing – original draft; project administration; writing – review and editing.

## Disclosure and competing interests statement

The authors declare that they have no conflict of interest.

## Supporting information



Appendix

Table EV1

Table EV2

Table EV3

Table EV4

Table EV5

Table EV6

Dataset EV1

Dataset EV2

Dataset EV3

Source Data for Figure 2

Source Data for Figure 3

Source Data for Figure 4

## Data Availability

Data supporting the findings of this work are available within the paper and its supplementary (Figures and Tables) files. The yeast strains of our collection are available upon request and after agreeing and signing the adequate MTA document. Raw sequences (fastq files; ITS‐amplicon data) from the dataset published by de Celis *et al* (2022), used to infer the population prevalence and relative abundance patterns of the yeast genera included in our collection, are available at NCBI Bioproject: PRJNA814622. The R codes and data sources used in this study are available in: https://github.com/Javier‐R‐Ruiz/Predicting‐wine‐yeast‐ecosystem‐function.
