# Supplementary material for: Predictability of the community‐function landscape in wine yeast ecosystems
Source: Mol Syst Biol. 2023 Aug 7;19(9):e11613. doi: 10.15252/msb.202311613 (PMC10495813; doi:10.15252/msb.202311613)
Supplement: Supplementary file 1 — Appendix [file MSB-19-e11613-s009.pdf]

Appendix

Ruiz & de Celis, et al. Predictability of the community-function landscape in wine yeast ecosystems.

Table of contents

Appendix Figure S1…page 2

Appendix Figure S2…page 3

Appendix Figure S3…page 4

Appendix Figure S4…page 5

Appendix Figure S5…page 6

Appendix Figure S6…page 7

Appendix Figure S7…page 8

Appendix Figure S8…page 9

Appendix Figure S9…page 10

Appendix Figure S10…page 11

Appendix Figure S11…page 12

Appendix Figure S12…page 13

Appendix Figure S13…page 14

Appendix Figure S14…page 15

Appendix Figure S15…page 16

Appendix Figure S16…page 17

Appendix Figure S17…page 18

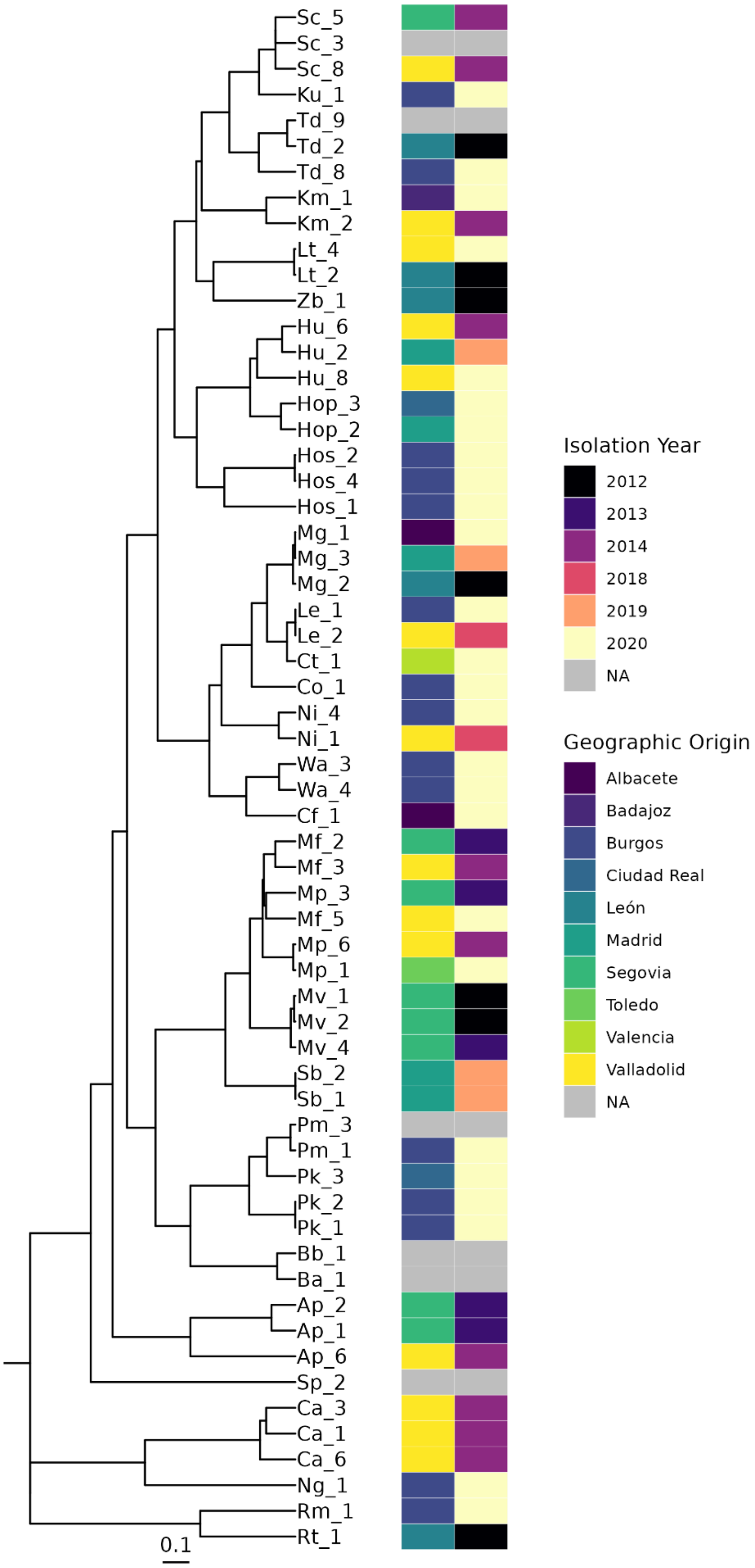

Appendix Figure S1. Phylogenetic relationships representation of the wine yeast strains used in this work. The phylogenetic tree was constructed using the 26s rRNA gene sequences of the strains (Table S1). The sequences aligned were used to construct a maximum likelihood phylogenetic tree. Consensus tree was rooted using the Basidiomycota group as an outgroup.

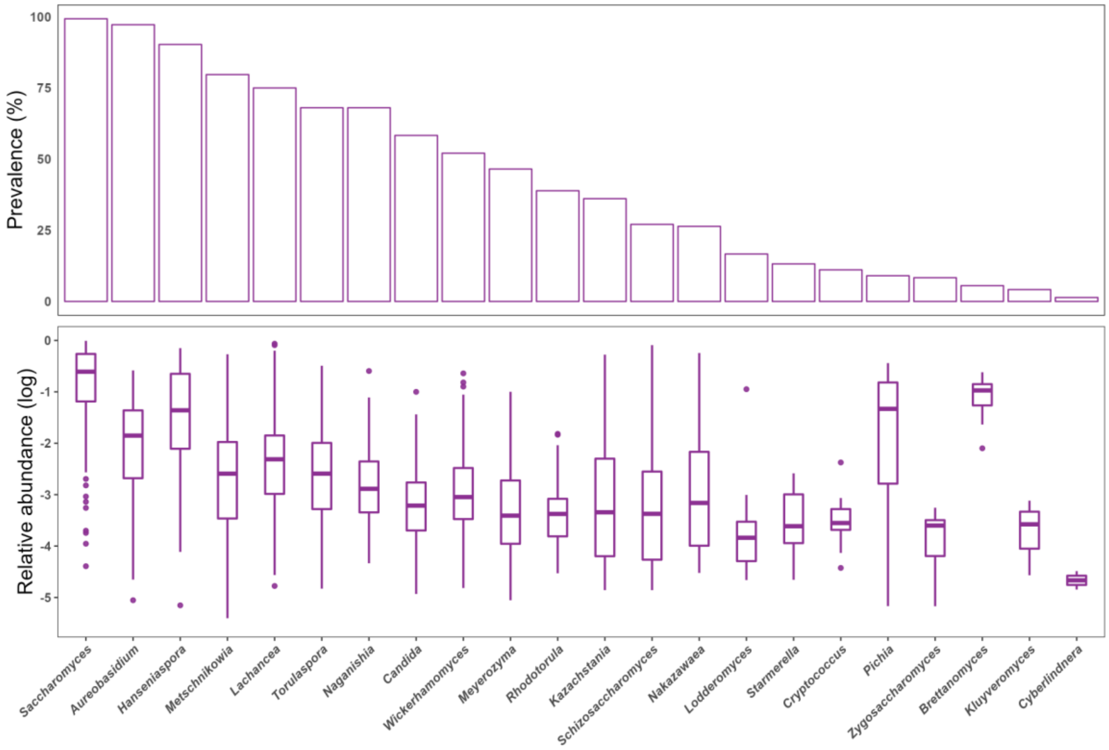

Appendix Figure S2. All the strains included in the collection of this work belong to yeast genera that are found, to a greater or lesser extent, in wine alcoholic fermentation. We show the estimated figures of population prevalence and relative abundance for the 22 yeast genera included in the collection, inferred using the ITS-amplicon data published by de Celis et al. (2022) from a large survey of 144 wine alcoholic fermentation samples. In the case of the genus *Metschnikowia*, data were obtained from Vicente et al. (2020).

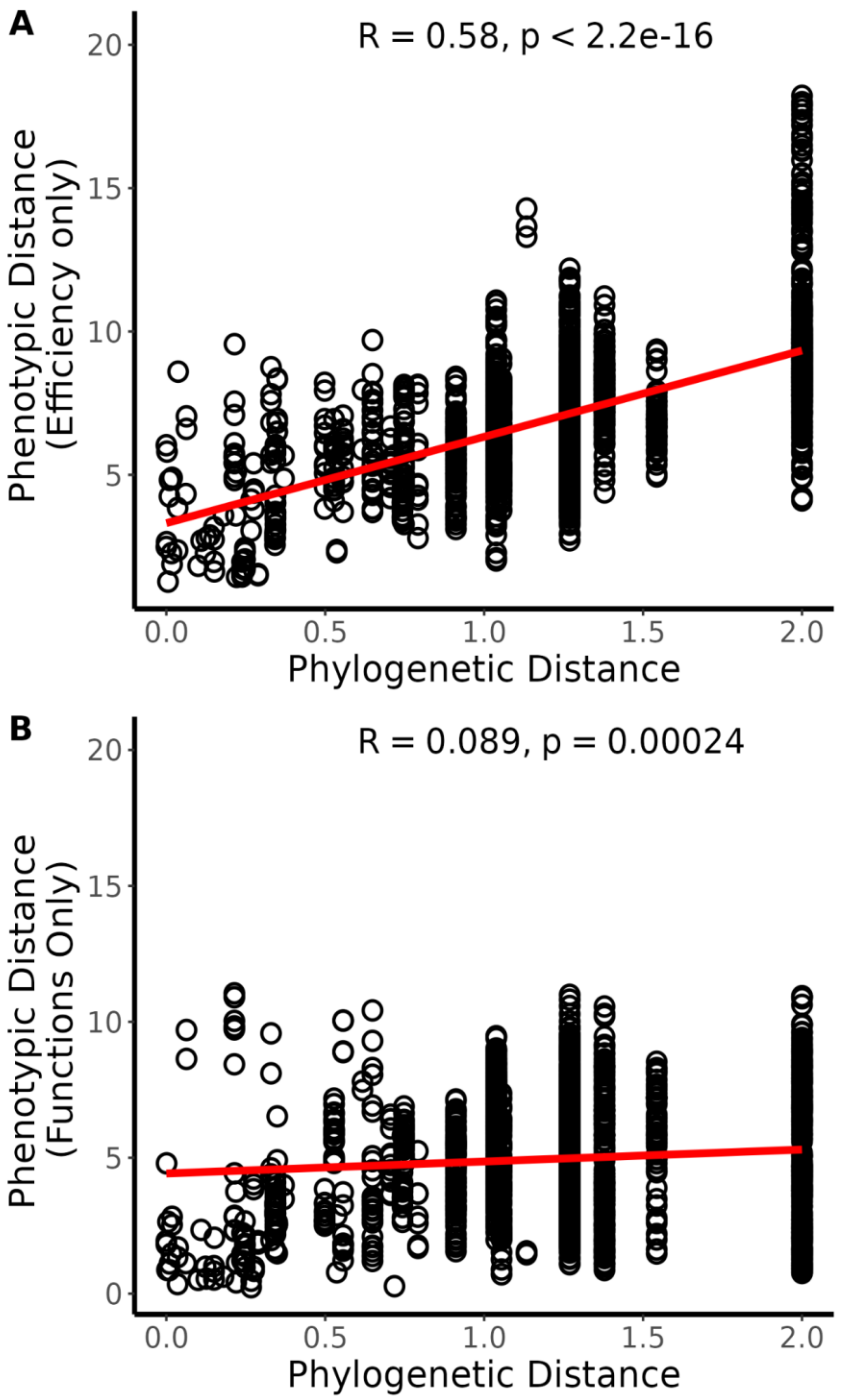

Appendix Figure S3. Phylogenetic distance among the strains significantly correlated to their phenotypic distance. Correlation between phylogenetic distance (the sum of branch lengths between strains pairs in the phylogenetic tree, Figure S1) and the phenotypic distance (Euclidean distance between the strains in the matrix of the phenotypic trait measured for each strain). (**A)** represents the phenotypic distance including just the wine physical-chemical parameters measured after 168h of growth in SGM (functions). (**B)** represents the phenotypic distance including just the efficiency growth value under the 28 environmental preferences assays. Dots represent each strain and red lines represent the Pearson correlation.

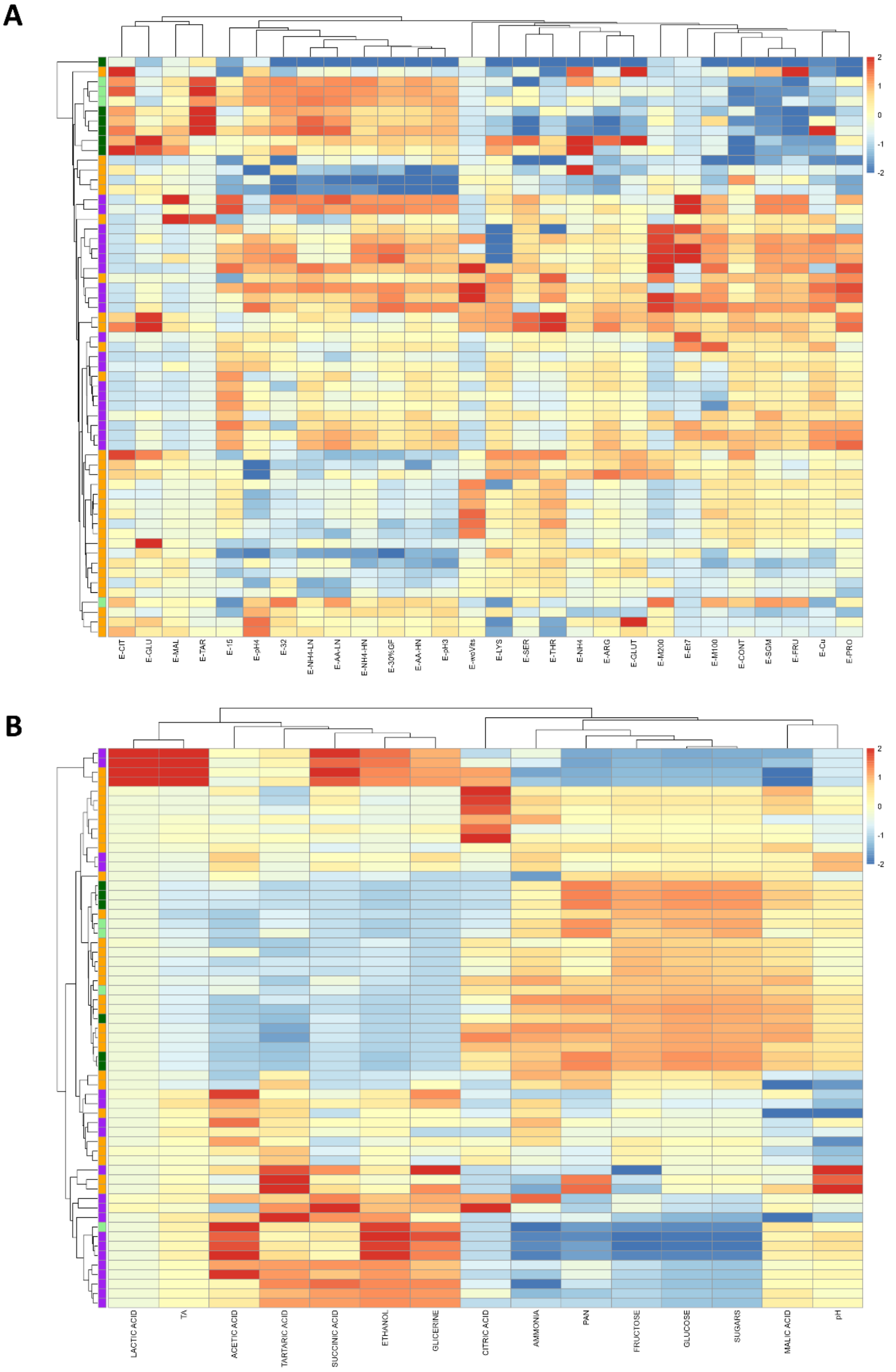

Appendix Figure S4. Phenotypic trait heatmaps for 60 wine yeast strains showing **(A)** their environmental preferences, as growth efficiency in 28 different growing conditions (Table EV2), and **(B)** their wine fermentation performance, measuring 15 wine physical-chemical parameters after 168h of fermentation in Synthetic Grape Must (SGM) (Table EV3). Trait values are centred and scaled for visualisation purposes. Colours to the left of the heatmap correspond to the lowest taxonomic level shared with *S. cerevisiae* (purple: same family, orange: same order, light green: same division, dark green: same kingdom. Strains are clustered based on their phenotypic behaviour rather than the phylogeny (Figure 2A & B).

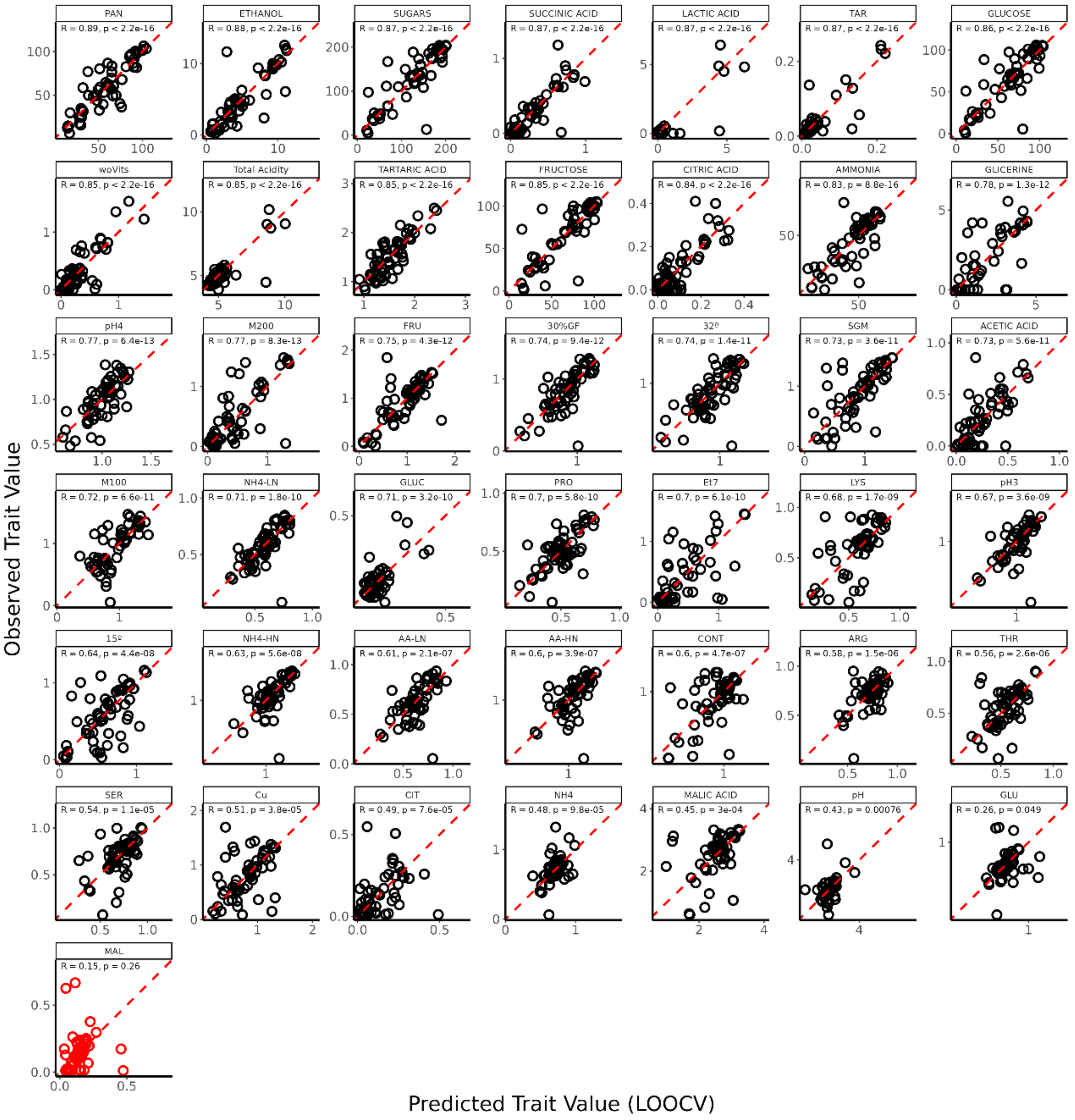

Appendix Figure S5. Most of the phenotypic traits in the wine yeast collection of the study are predictable using the phylogeny. Panel of predicted vs observed traits (28 environmental preferences analysed and 15 wine related parameters analysed after Synthetic Grape Must fermentation). Just the efficiency growth using malic acid as sole carbon source (MAL), coloured in red, was not accurately predicted based on the phylogeny (R=0.15, p=0.26). Predictions were made using phylogenetic imputation and tested by the leave one out-cross validation (LOOCV).

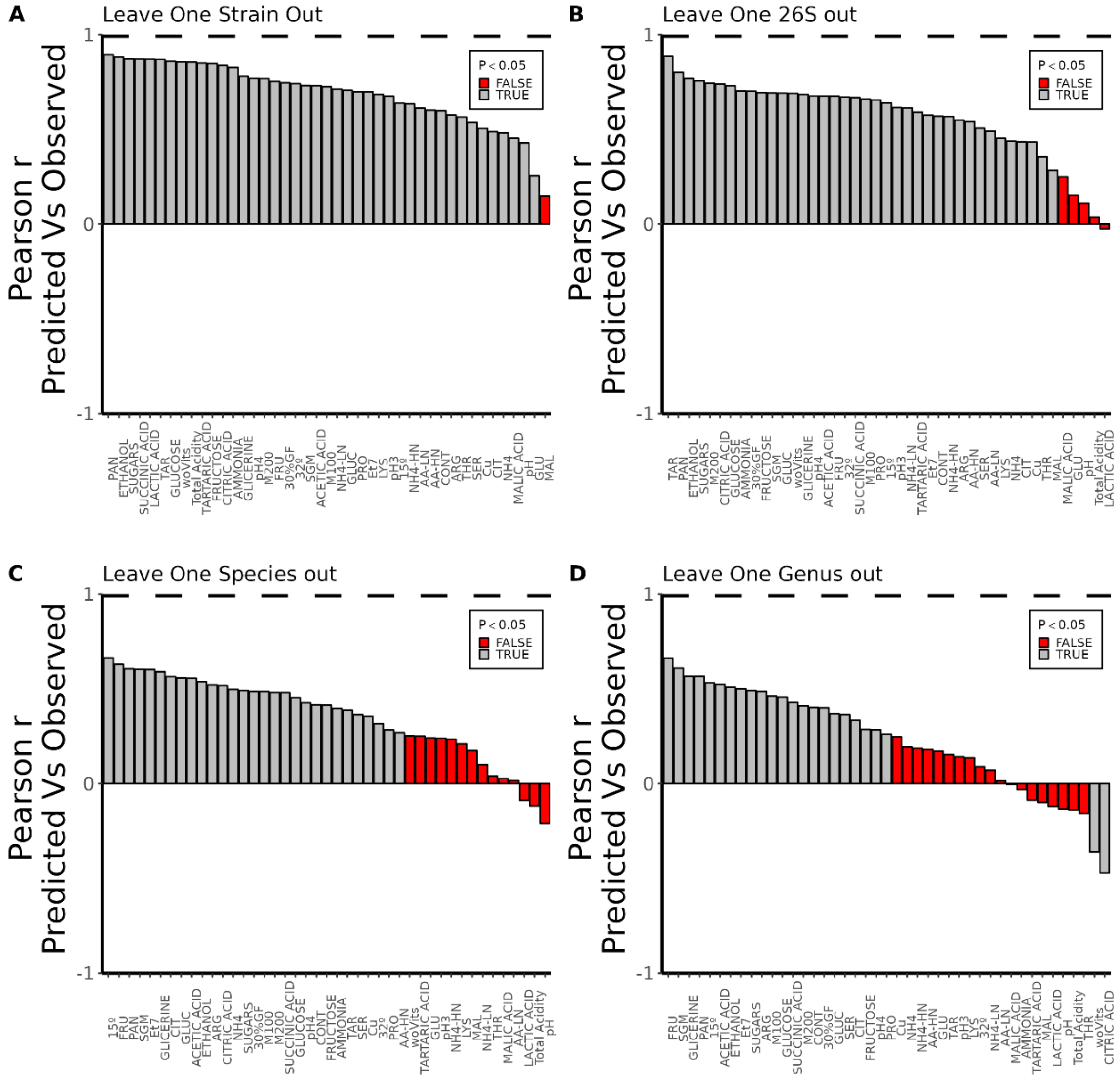

Appendix Figure S6. **Robustness testing of the phylogenetic imputation based on the Leave-One-Out Cross-Validation (LOOCV) method.** We performed the LOOCV method at different taxonomic levels to test how the presence of different strains with low phylogenetic distance affect the phenotype imputation. We show correlation between predicted and observed traits values for all 43 traits. The panels represent the correlation coefficients obtained from the LOOCV method by **A** using all the strains of the collection, **B** only using the strains with different 26S sequence (removing the phylogenetic identical strains), **C** only using the strains belonging to different species and **D** only using strains belonging to different genera.

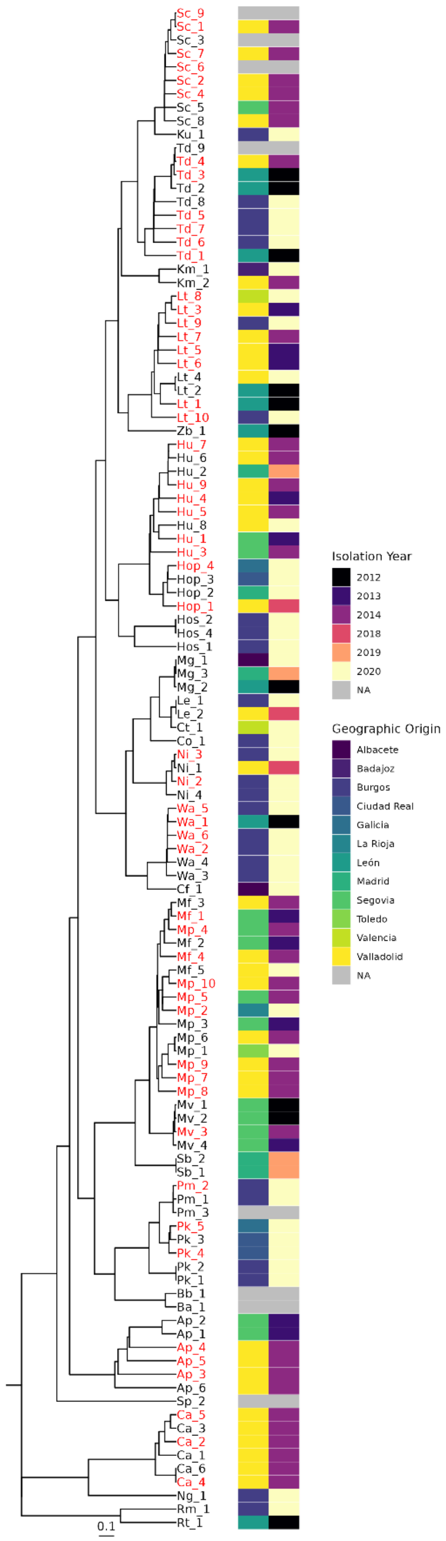

Appendix Figure S7. Phylogenetic relationships representation of the 113 yeast strains. The phylogenetic tree was constructed using the 26s rRNA gene sequences of the 113 strains (60 strains from the original collection, shown in Table S1 and 53 strains from a new collection shown in Table S5). The sequences aligned were used to construct a maximum likelihood phylogenetic tree. Consensus tree was rooted using the Basidiomycota group as an outgroup. Red colour indicates the 53 strains from the new set of strains used to check the accuracy of phylogenetically based phenotype predictions.

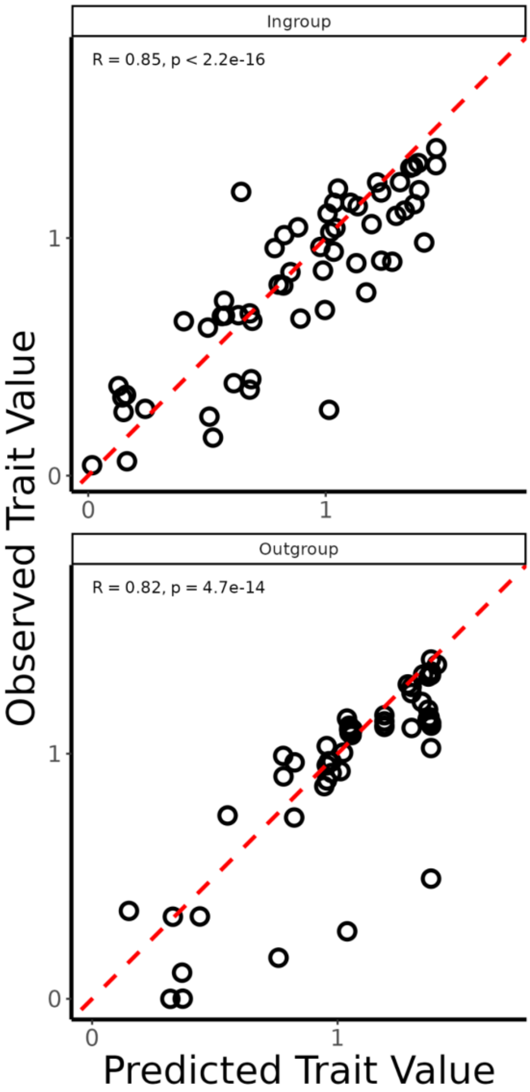

Appendix Figure S8. The prediction based on phylogeny allows to predict the phenotype of new strains with a similar accuracy as replicate measures. To check the accuracy of our phylogenetically based prediction model, we assayed the growth efficiency in SGM of a wider collection of strains (the 60 strains already characterised and 53 new strains (Table S6)). In this way, we characterised this trait in 60 strains in two independent batches of experiments. The upper plot represents the prediction of this trait in the 60 strains based on the efficiency values of the second batch of the experiment. The lower plot represents the prediction of the 60 strains based on the phylogenetic relationships of the 53 new strains.

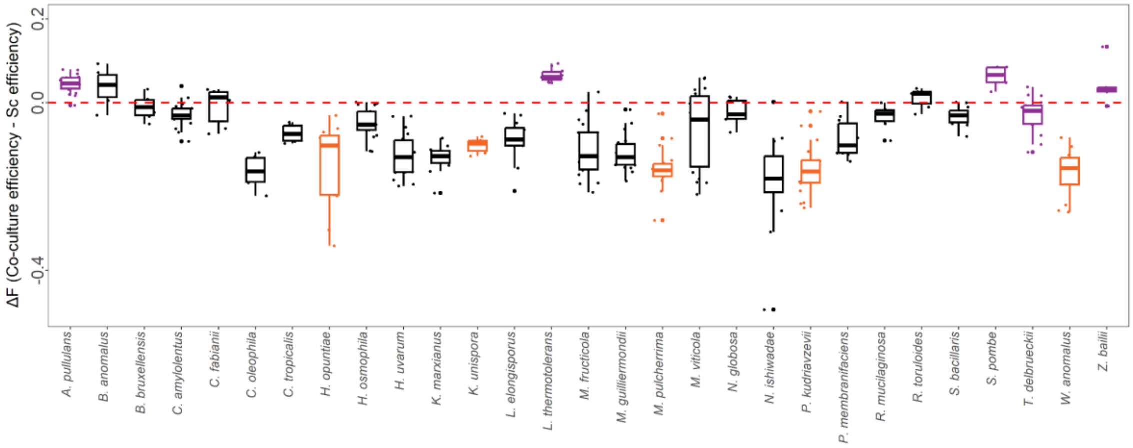

Appendix Figure S9. Some wine yeast species cause a negative impact on the growth efficiency of *S. cerevisiae*. To explore the effect of non-*Saccharomyces* strains on *S. cerevisiae* growth, we co-inoculated each non-*Saccharomyces* strain with *S. cerevisiae* Sc_5 strain. The functional effect (ΔF) of the different species were calculated by comparing efficiency values (total variation in cells density) of the co-cultures and the efficiency values of *S. cerevisiae* individual culture (Sc_5xSc_5), after 168h of SGM fermentation (Table S7). Purple and orange colours represent those species from which we selected strains with an enhanced or negative effect on *S. cerevisiae* co-culture performance, respectively. Boxplots represent the median and standard deviation of all the replicates of the different strains belonging to the same species.

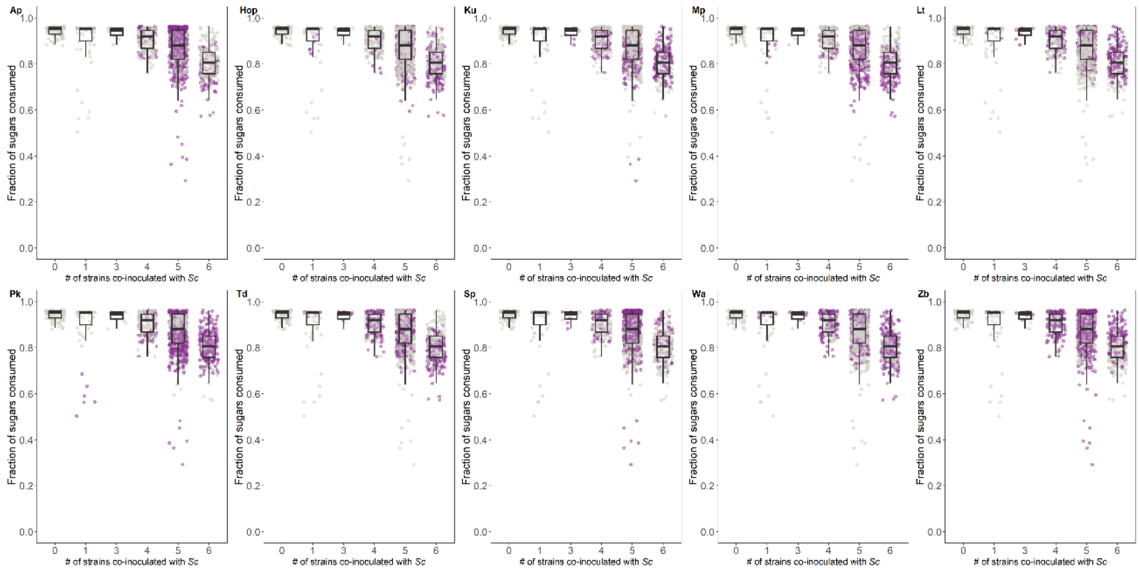

Appendix Figure S10. Some strains tend to be preferentially distributed in low-functioning communities. We represent how the increase of richness of the background community (containing *S. cerevisiae*) increases the chances to detect negative interactions on the community function. Each dot represents the fraction of sugars consumed of each community in which *S. cerevisiae* strains were inoculated. Coloured dots in every plot represent the communities that contain every specific strain (represented in the different panels).

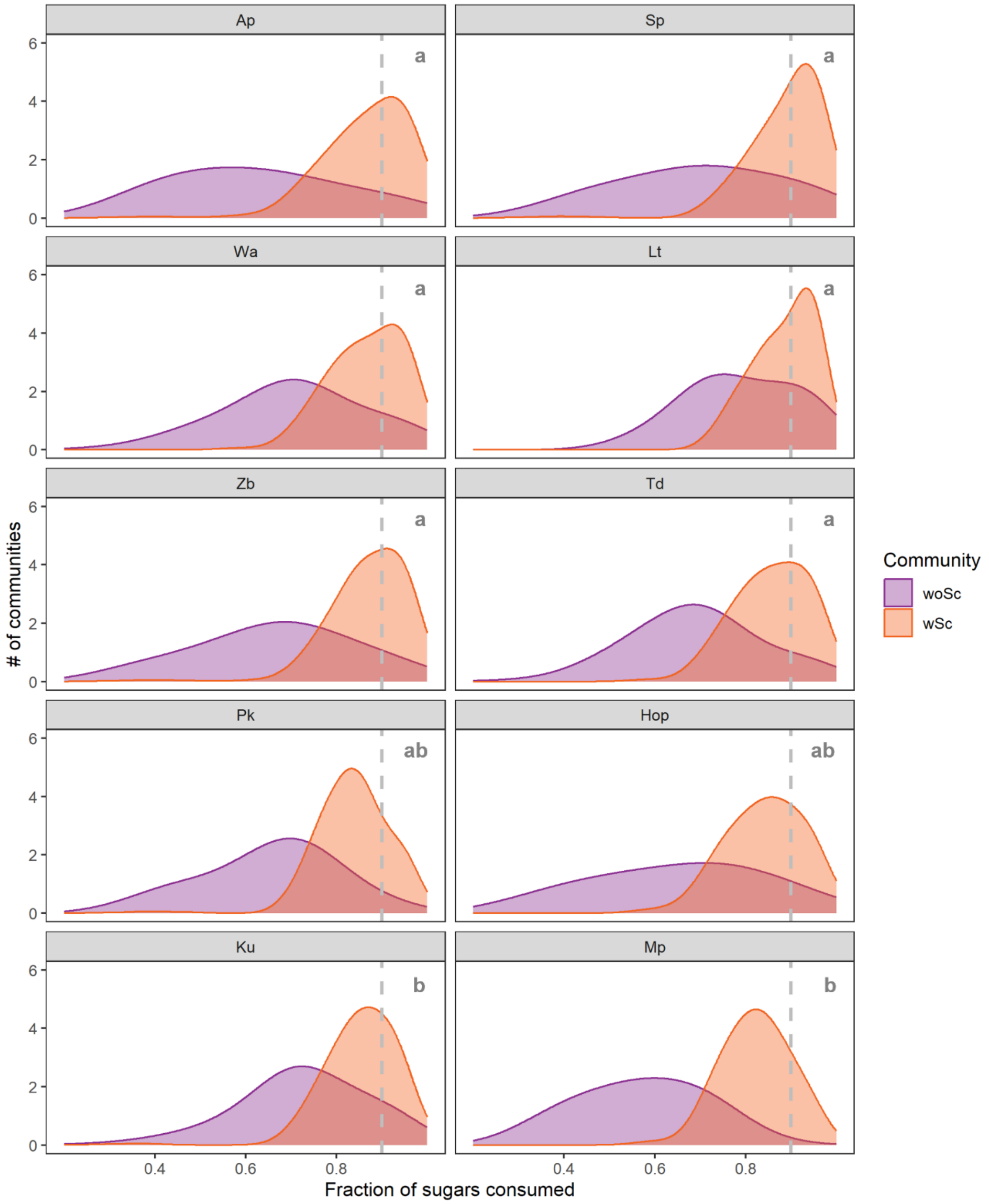

Appendix Figure S11. Strains were not equally distributed among the communities based on their ecological function. We found that some strains tend to be distributed in communities with lower function (less than the 70-60% of the sugars consumed after the fermentation) and other strains tend to be distributed in higher function communities (more than the 80-90% of the sugars consumed). Colours indicate the distribution of the communities that contain a *S. cerevisiae* strain (orange) and communities that do not contain a *S. cerevisiae* strain (purple). Strains are ordered in the panel according to the number of *S. cerevisiae*-containing communities that consumed more than 90% of the sugars (dashed lines in the plots). Different letters indicate the existence of statistical differences in the strains based on their presence in high-functioning communities, i.e., communities that consume at least the 90% of the sugars (ANOVA test, $p<0.05$).

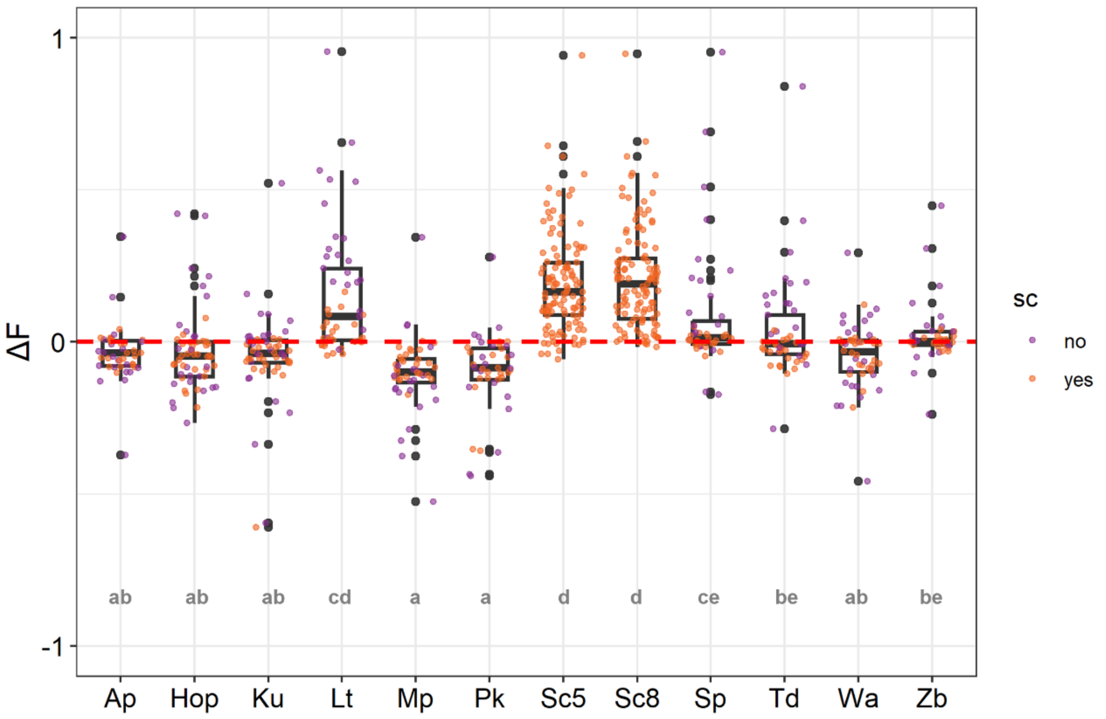

Appendix Figure S12. Some strains consistently exert a detrimental impact on community function. We represent the functional effect (ΔF) of the different strains assayed against the different background communities. The ΔF values were calculated by comparing the sugars consumption value of the community (background community + strain co-inoculated) and the sugars consumption value of the background community. Orange and purple colours represent if the community harbours or not a *S. cerevisiae* strain, respectively. Boxplots represent the median and standard deviation of all the communities where these species were co-inoculated. Different letters indicate the existence of statistical differences among the ΔF values of the different strains (ANOVA, $p<0.05$).

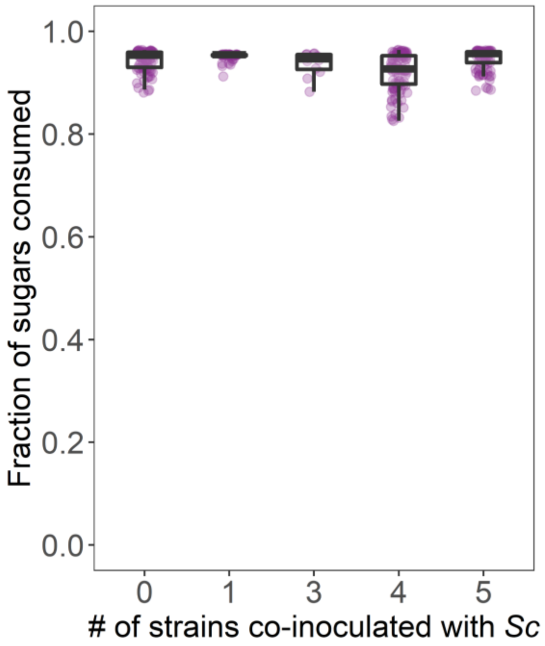

Appendix Figure S13. Community diversity does not affect function in communities that harbours neutral-effect strains. We just represented the communities that do not contain Mp, Pk and Hop strains (strains that cause a negative effect in the function of co-cultures with *S. cerevisiae*, Figure 3D). Each dot represents the fraction of sugars consumed by each community in which *S. cerevisiae* strains were inoculated.

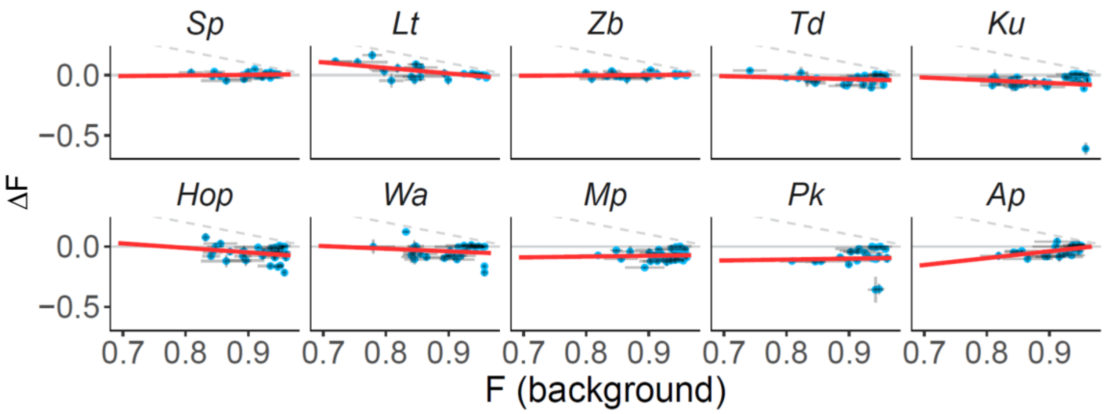

Appendix Figure S14. Functional Effect Equations of the strains on yeast communities that contain *S. cerevisiae*. When we explore the FEEs of the strains just considering communities that contain *S. cerevisiae,* the clear patterns of the strains observed in Figure 4B are lost (as *S. cerevisiae* is the main contributor to the ecological function of wine fermentation). However, some strains (mainly *Mp* and *Pk*, and to a lesser extent, *Hop* and *Ap*) still show marked negative patterns in the function of the communities regardless of the function of the background community.

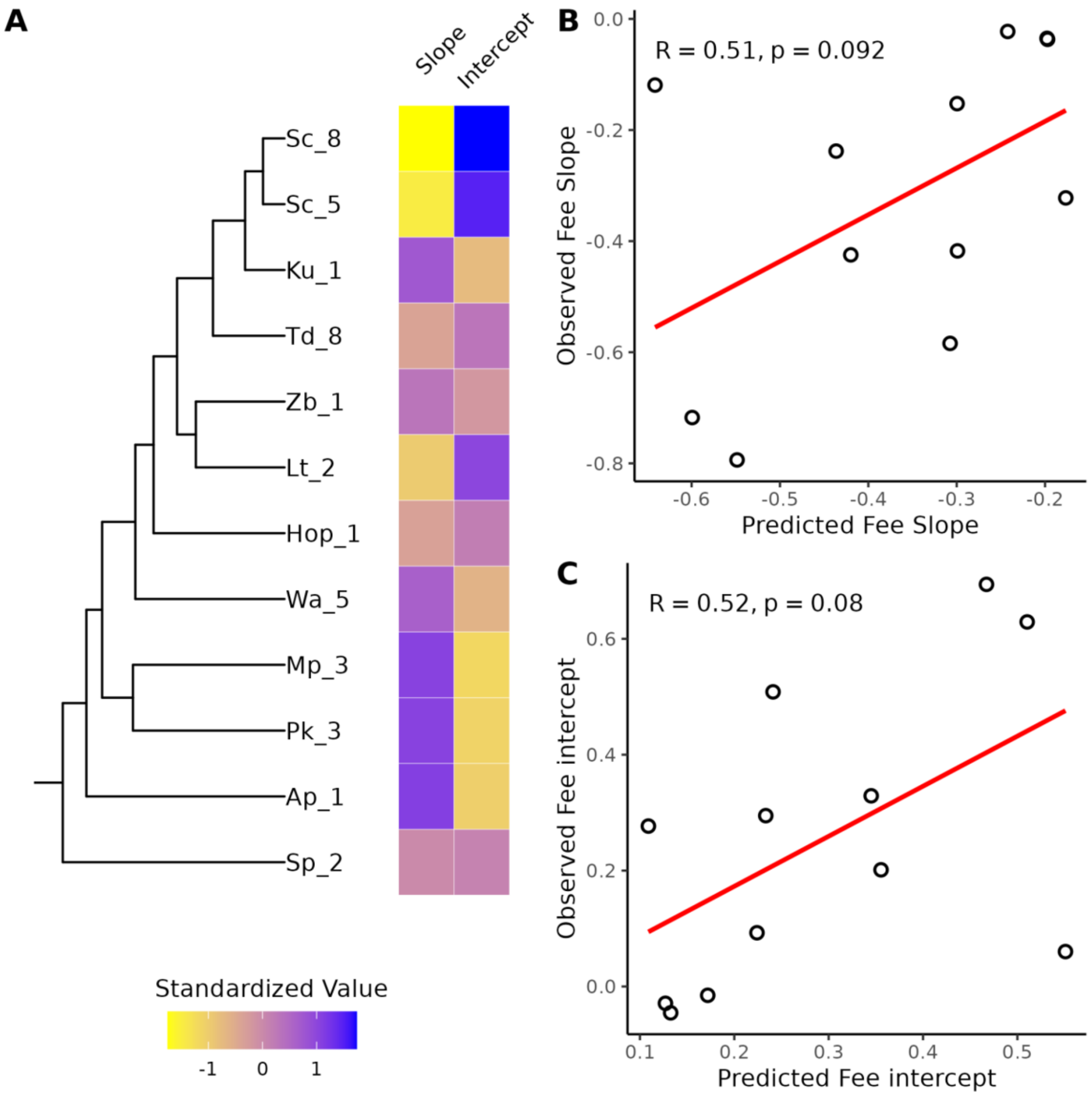

Appendix Figure S15. Functional effects of the strains on their ecological context (FEEs slopes and intercepts) are less predictable from phylogeny than the individual phenotypic traits. The phylogenetic tree and heatmap representation (**A**) show that the mathematical parameters that define the ecological effect of the strains in the wine yeasts communities (FEEs slope and intercept) can be clustered based on the phylogenetic relationships of the strains**.** Also, some correlation signal is observed between the observed and the predicted based on phylogeny FEEs parameters: slope (**B**) and intercept (**C**). However, these correlations are not significative, showing that the functional effect of strains cannot be clearly predicted using the phylogeny imputation. The strong impact of the function of the background communities might explain this loss of predictability when we consider the ecological effect of the strains.

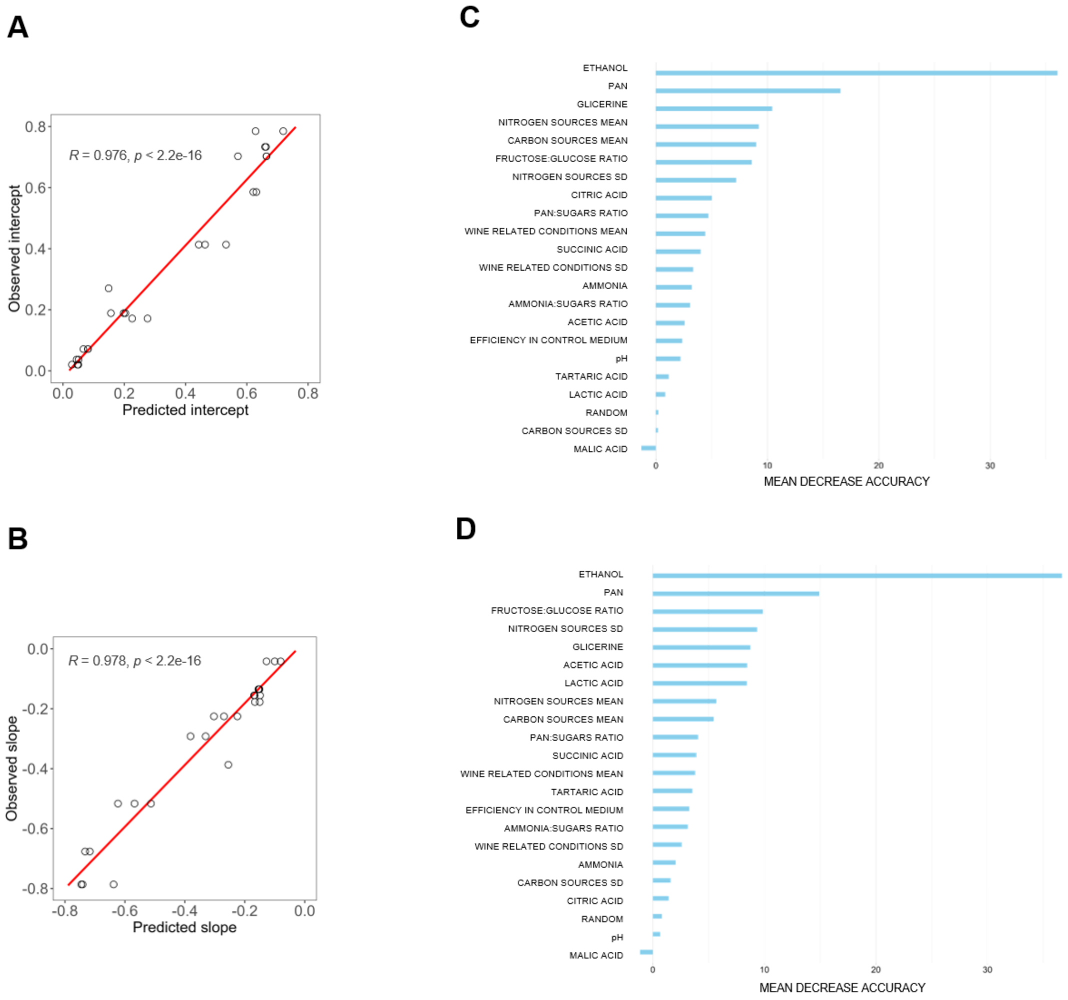

Appendix Figure S16. Ethanol production and Primary Amino acid Nitrogen consumption are the phenotypic traits that better explain the ecological effect of the strains in wine yeast communities. A random forest model was used to predict the FEEs parameters; intercept **(A)** and slope **(B)** based on the 43 phenotypic traits analysed in this study (Figure 2A-B). The percentage of model accuracy that decreases when we leave out each trait is represented for the intercept prediction **(C)** and for the slope prediction **(D)**.

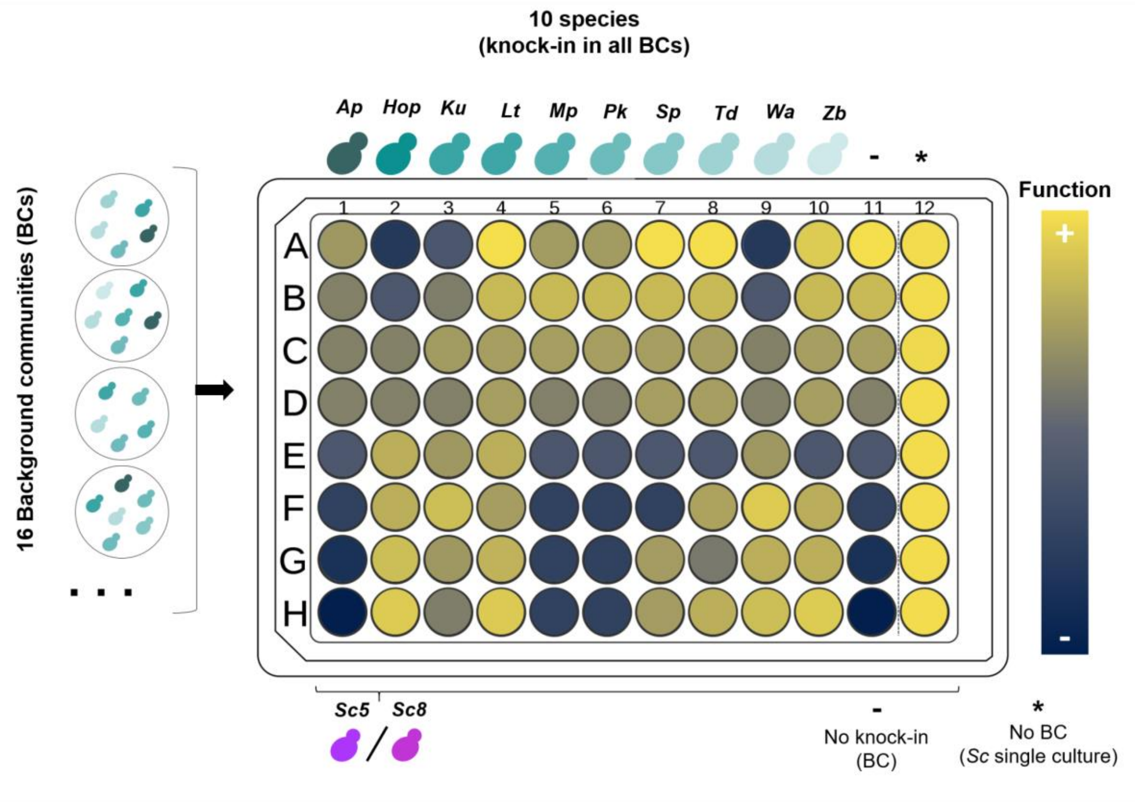

Appendix Figure S17. Experimental design for the study of ecological effect of strains on different yeast background communities. A total of 16 different background communities (composed with 3, 4, 5 or 6 non-*Saccharomyces* strains) were inoculated in Synthetic Grape Must on 96-well plates. In addition, every single strain was co-inoculated in the wells containing each background community, in order to increase the number of possible combinations of strains. Three plates with this display were prepared; one plate was inoculated with Sc5 *S. cerevisiae* strain, another plate was inoculated with Sc8 *S. cerevisiae* strain, and another plate was not inoculated with any *S. cerevisiae* strain. Thus, we created a total of 528 different communities, inoculated by triplicate. After 168h of fermentation, the ecological function of each community was calculated by measuring the residual sugars (glucose +fructose). Yellow colour indicates high function communities (a high fraction of sugars was consumed after fermentation), blue colour indicates low function communities (a low fraction of sugars was consumed after fermentation). The functions of all the communities, pairwise and individual assays are shown in Table S8.
