# Supplementary material for: Predictability of the community‐function landscape in wine yeast ecosystems
Source: Mol Syst Biol. 2023 Aug 7;19(9):e11613. doi: 10.15252/msb.202311613 (PMC10495813; doi:10.15252/msb.202311613)
Supplement: Supplementary file 14 [file MSB-19-e11613-s013.pdf]

Predictability of the community-function landscape in wine yeast ecosystems

Javier Ruiz, Miguel de Celis, Juan Diaz-Colunga, Jean Vila, Belen Benitez-Dominguez, Javier Vicente, Antonio Santos, Alvaro Sanchez, and Ignacio Belda
DOI: 10.15252/msb.202311613

Corresponding author(s): Ignacio Belda (ignaciobelda@ucm.es)

Review Timeline:

Submission Date:	21st Feb 23
Editorial Decision:	22nd Mar 23
Revision Received:	12th Jun 23
Editorial Decision:	24th Jul 23
Revision Received:	26th Jul 23
Accepted:	26th Jul 23

Editor: Maria Polychronidou/Jingyi Hou

Transaction Report:

(Note: With the exception of the correction of typographical or spelling errors that could be a source of ambiguity, letters and reports are not edited. Depending on transfer agreements, referee reports obtained elsewhere may or may not be included in this compilation. Referee reports are anonymous unless the Referee chooses to sign their reports.)

22nd Mar 2023

Manuscript Number: MSB-2023-11613
Title: Predictability of the community-function landscape in wine yeast ecosystems
Author: Javier Ruiz
Miguel de Celis
Juan Diaz-Colunga
Jean Vila
Belen Benitez-Dominguez
Javier Vicente
Antonio Santos
Alvaro Sanchez
Ignacio Belda

Dear Dr Belda,

Thank you for submitting your work to Molecular Systems Biology. We have now heard back from the three reviewers who agreed to evaluate your manuscript. As you will see from the reports below, the reviewers acknowledge the potential interest of the study. They raise however a series of concerns, which we would ask you to address in a major revision.

I think the recommendations of the reviewers are rather clear, so there is no need to repeat the points listed below. In particular, Reviewer #1 was concerned about the level of physiological and mechanistic insight provided and pointed out that several conclusions were overly generalized. We would ask you to address these concerns by providing a thorough discussion and a better contextualization for your claims. The issue of using a single marker gene should be discussed as well.

All other issues raised by the reviewers need to be satisfactorily addressed. As you may already know, our editorial policy allows in principle a single round of major revision, and it is therefore essential to provide responses to the reviewers' comments that are as complete as possible. Please feel free to contact me in case you would like to discuss in further detail any of the issues raised by the reviewers.

On a more editorial level, we would ask you to address the following issues:

- Please provide a .docx formatted version of the manuscript text (including legends for main figures, EV figures and tables). Please make sure that the changes are highlighted to be clearly visible.

- Please provide individual production quality figure files as .eps, .tif, .jpg (one file per figure).

-Please provide a .docx formatted letter INCLUDING the reviewers' reports and your detailed point-by-point responses to their comments. As part of the EMBO Press transparent editorial process, the point-by-point response is part of the Review Process File (RPF), which will be published alongside your paper.

-Please note that all corresponding authors are required to supply an ORCID ID for their name upon submission of a revised manuscript.

-We replaced Supplementary Information with Expanded View (EV) Figures and Tables that are collapsible/expandable online (see examples in http://msb.embopress.org/content/11/6/812). A maximum of 5 EV Figures can be typeset. EV Figures should be cited as 'Figure EV1, Figure EV2" etc... in the text and their respective legends should be included in the main text after the legends of regular figures.

Additional Tables/Datasets should be labeled and referred to as Table EV1, Dataset EV1, etc. Legends have to be provided in a separate tab in case of .xls files. Alternatively, the legend can be supplied as a separate text file (README) and zipped together with the Table/Dataset file.

For the figures and tables that you do NOT wish to display as Expanded View figures, they should be bundled together with their legends in a single PDF file called *Appendix*, which should start with a short Table of Content. Each legend should be below the corresponding Figure/Table in the Appendix. Appendix figures and tables should be referred to in the main text as: "Appendix Figure S1, Appendix Figure S2, Appendix Table S1" etc. See detailed instructions regarding expanded view here: https://www.embopress.org/page/journal/17444292/authorguide#expandedview.

-Before submitting your revision, primary datasets (and computer code, where appropriate) produced in this study need to be deposited in an appropriate public database (seehttp://msb.embopress.org/authorguide - dataavailability

https://www.embopress.org/page/journal/17444292/authorguide#dataavailability).

Please remember to provide a reviewer password if the datasets are not yet public.

The accession numbers and database should be listed in a formal "Data Availability" section (placed after Materials & Method) that follows the model below (see also https://www.embopress.org/page/journal/17444292/authorguide#dataavailability). Please note that the Data Availability Section is restricted to new primary data that are part of this study.

Data availability

The datasets (and computer code) produced in this study are available in the following databases:

- RNA-Seq data: Gene Expression Omnibus GSE46843 (https://www.ncbi.nlm.nih.gov/geo/query/acc.cgi?acc=GSE46843)
- [data type]: [name of the resource] [accession number/identifier/doi] ([URL or identifiers.org/DATABASE:ACCESSION])

*** Note - All links should resolve to a page where the data can be accessed. ***

-At EMBO Press we ask authors to provide source data for the main figures. Our source data coordinator will contact you to discuss which figure panels we would need source data for and will also provide you with helpful tips on how to upload and organize the files.

- Our journal encourages inclusion of *data citations in the reference list* to directly cite datasets that were re-used and obtained from public databases. Data citations in the article text are distinct from normal bibliographical citations and should directly link to the database records from which the data can be accessed. In the main text, data citations are formatted as follows: "Data ref: Smith et al, 2001". In the Reference list, data citations must be labeled with "[DATASET]". A data reference must provide the database name, accession number/identifiers and a resolvable link to the landing page from which the data can be accessed at the end of the reference. Further instructions are available at .

- We updated our journal's competing interests policy in January 2022 and request authors to consider both actual and perceived competing interests. Please review the policy https://www.embopress.org/competing-interests and update your competing interests if necessary.

Please use the heading "Disclosure statement and competing interests".

- All Materials and Methods need to be described in the main text. We would encourage you to use 'Structured Methods', our new Materials and Methods format. According to this format, the Material and Methods section should include a Reagents and Tools Table (listing key reagents, experimental models, software and relevant equipment and including their sources and relevant identifiers) followed by a Methods and Protocols section in which we encourage the authors to describe their methods using a step-by-step protocol format with bullet points, to facilitate the adoption of the methodologies across labs. More information on how to adhere to this format as well as downloadable templates (.doc or .xls) for the Reagents and Tools Table can be found in our author guidelines: < https://www.embopress.org/page/journal/17444292/authorguide#researcharticleguide>. An example of a Method paper with Structured Methods can be found here: .

-Regarding data quantification:
Please ensure to specify the name of the statistical test used to generate error bars and P values, the number (n) of independent experiments (please specify technical or biological replicates) underlying each data point and the test used to calculate p-values in each figure legend. Discussion of statistical methodology can be reported in the materials and methods section, but figure legends should contain a basic description of n, P and the test applied.
Graphs must include a description of the bars and the error bars (s.d., s.e.m.).
Please also include scale bars in all microscopy images.

- Please provide a "standfirst text" summarizing the study in one or two sentences (approximately 250 characters, including space), three to four "bullet points" highlighting the main findings and a "synopsis image" (550px width and 400-600 px height, PNG format) to highlight the paper on our homepage.
Here are a couple of examples:
https://www.embopress.org/doi/10.15252/msb.20199356
https://www.embopress.org/doi/10.15252/msb.20209475
https://www.embopress.org/doi/10.15252/msb.209495

When you resubmit your manuscript, please download our CHECKLIST (https://www.embopress.org/pb-assets/embo-site/EMBO%20Press%20Author%20Checklist-1642513524327.xlsx) and include the completed form in your submission. *Please note* that the Author Checklist will be published alongside the paper as part of the transparent process (https://www.embopress.org/page/journal/17444292/authorguide#transparentprocess).

If you feel you can satisfactorily deal with these points and those listed by the referees, you may wish to submit a revised version of your manuscript. Please attach a covering letter giving details of the way in which you have handled each of the points raised by the referees. A revised manuscript will be once again subject to review and you probably understand that we can give you no guarantee at this stage that the eventual outcome will be favorable.

We look forward to receiving your revised manuscript soon.

Sincerely,
Jingyi

Jingyi Hou, PhD
Scientific Editor
Molecular Systems Biology

We realize that it is difficult to revise to a specific deadline. In the interest of protecting the conceptual advance provided by the work, we recommend a revision within 3 months (20th Jun 2023). Please discuss the revision progress ahead of this time with the editor if you require more time to complete the revisions. Use the link below to submit your revision:

https://msb.msubmit.net/cgi-bin/main.plex

IMPORTANT: When you send your revision, we will require the following items:
1. the manuscript text in LaTeX, RTF or MS Word format
2. a letter with a detailed description of the changes made in response to the referees. Please specify clearly the exact places in the text (pages and paragraphs) where each change has been made in response to each specific comment given
3. three to four 'bullet points' highlighting the main findings of your study
4. a short 'blurb' text summarizing in two sentences the study (max. 250 characters)
5. a 'thumbnail image' (550px width and max 400px height, Illustrator, PowerPoint or jpeg format), which can be used as 'visual title' for the synopsis section of your paper.
6. Please include an author contributions statement after the Acknowledgements section (see https://www.embopress.org/page/journal/17444292/authorguide)
7. Please complete the CHECKLIST available at (https://bit.ly/EMBOPressAuthorChecklist).
Please note that the Author Checklist will be published alongside the paper as part of the transparent process (https://www.embopress.org/page/journal/17444292/authorguide#transparentprocess).
8. When assembling figures, please refer to our figure preparation guideline in order to ensure proper formatting and readability in print as well as on screen:
https://bit.ly/EMBOPressFigurePreparationGuideline
See also figure legend guidelines: https://www.embopress.org/page/journal/17444292/authorguide#figureformat
9. Please note that corresponding authors are required to supply an ORCID ID for their name upon submission of a revised manuscript (EMBO Press signed a joint statement to encourage ORCID adoption).
(https://www.embopress.org/page/journal/17444292/authorguide#editorialprocess)
Currently, our records indicate that the ORCID for your account is 0000-0002-2607-5049.

Please click the link below to modify this ORCID:
Link Not Available

10. At EMBO Press we ask authors to provide source data for the main manuscript figures. Our source data coordinator will contact you to discuss which figure panels we would need source data for and will also provide you with helpful tips on how to upload and organize the files.

The system will prompt you to fill in your funding and payment information. This will allow Wiley to send you a quote for the article processing charge (APC) in case of acceptance. This quote takes into account any reduction or fee waivers that you may be eligible for. Authors do not need to pay any fees before their manuscript is accepted and transferred to the publisher.

EMBO Press participates in many Publish and Read agreements that allow authors to publish Open Access with reduced/no publication charges. Check your eligibility: https://authorservices.wiley.com/author-resources/Journal-Authors/open-access/affiliation-policies-payments/index.html

As a matter of course, please make sure that you have correctly followed the instructions for authors as given on the submission website.

*** PLEASE NOTE *** As part of the EMBO Press transparent editorial process initiative (see our Editorial at https://dx.doi.org/10.1038/msb.2010.72), Molecular Systems Biology publishes online a Review Process File with each accepted manuscripts. This file will be published in conjunction with your paper and will include the anonymous referee reports, your point-by-point response and all pertinent correspondence relating to the manuscript. If you do NOT want this File to be published, please inform the editorial office at msb@embo.org within 14 days upon receipt of the present letter.

Reviewer #1:

This study aims at inferring microbial community (mixtures of wine yeast isolates) phenotypes related to wine fermentation by using a linear model (described in another publication) combining phylogenetic relatedness and individual species/strain phenotyping data. The phylogenetic relatedness is based on a single marker gene (26S rRNA) and phenotype defined using sugar consumption and growth/secretion parameters relevant for wine fermentation. The study uses 60 yeasts isolated from wine fermentation. While the overall concept is interesting and has possible biotechnological applications, there are several points where the study lacks evidence to support the conclusions drawn. There is neither concrete mechanistic insights into inter-strain interactions, nor any physiological insight into community-scale fermentation. Further, the genomic resolution is poor due to usage of single marker gene (as evident from Suppl Fig 1, where some of the isolates are identical). The conclusions drawn are thus not likely to be generalisable. Indeed, high-resolution studies in yeast (e.g., 1000 yeast project) have clearly shown that even genome sequencing is not sufficient to unravel complex genotype-phenotype relations in yeast.
The abstract, introduction, and discussion sections are overly generalized and also poorly contextualised (e.g., there is a single reference to 8 year old study for fermented food as model system and there is no explanation on in what way wine yeast community studies here makes a model - especially given no interaction network).
Below are some additional points that we hope will be useful.

1) The 60 yeast strains: Have these been obtained from the same fermentation environment? Can this be annotated? Following this, have the 53 strains that the authors phenotyped later in the first paragraph of the results been obtained from the same wine fermentations? By looking at the NCBI entries I understood that several of the strains were already isolated from previous works (e.g. Unraveling the Enzymatic Basis of Wine 'Flavorome': APhylo-Functional Study of Wine Related Yeast Species JOURNAL Front Microbiol 7, 12 (2016))
This information is vital because evolutionary processes such as population bottlenecks and adaptation to specific environmental niches, even different wine fermentations, as some of the authors point out in the mentioned publication above, can generate high phenotypic diversity in strains belonging to the same species. This would pose a challenge to inferring trait relatedness only using 26S as molecular marker though the signal may still be strong but it is not clear if that has been explored.

2) branch length in the phylogenetic tree in figure S1?

3) There are several mistakes in Table S1 at the annotation of the Family level. For instance, Brettanomyces isolates, Pichia isolates and Meyerozyma guillermondii isolates are annotated as family Saccharomycetacae but they do not belong to this family as the author showed in Figure 1B. Also, in legend of figure 1B is missing the explanation of the triangles at the end of the branches.

4) There is a mistake in the text when the authors report "Ascomycetes yeasts (purple and orange) exhibit, on average, higher growth efficiencies than basidiomycetes (light and dark green) (T-test, p=5.387e-07). Among Ascomycetes, Saccharomycetaceae strains (purple) have, on average, higher growth efficiencies than the non- Saccharomycetaceae strains (orange and light green) (T-test, p=1.604e-12)"
Light green refers to Basidiomycetes according to the legend provided by the authors, so the second paragraph should not include the "light green" strains in the comparison as those are basidiomycetes. Also please revise the use of italics across the manuscript, in some passages is missing.

5) In both Figure 2A and 2B the phylogenetic tree on the left is not aligned with the respective heatmap. Also, how the clustering using the phenotypic data looks like?

6) In figure S3 A the linear correlation is negligible R is less then 0.2 I would refrain to make strong affirmations given also the distribution of the points in the plot.

7) In the legend of Figure 2A "...indicates that the trait distribution amongst strains is highly influenced by their shared evolutionary history." I would avoid using the term "shared" with "evolutionary histories" in this context and maintain only "evolutionary history". The correlation implies that the phenotypic distance and the phylogenetic distance have a non-random relationship but it does not imply that the strains have "shared" evolutionary histories.

8) In Figure 2E the authors used the "Leave one out cross validation (LOOCV)" to validate their phenotypic imputation based on the phylogeny. I wondered if performing the same analysis by leaving out all but one strains from each phylogenetic unit at the species level would decrease the robustness of the correlation of the prediction versus the measured values. As phylopar re-roots to the closer common ancestor there are many cases where the LOOCV would use the sister strains from the same species with very low phylogenetic distance for re-rooting and impute a phenotype. This aspect is also pointed out by the authors "Predictions are also better at shorter phylogenetic distances, when there are closer relatives present in the tree" and I think it could be worth exploring it to assess the limit of the approach.

9) Referring to point 1) It would help to clarify where the 53 strains have been obtained and what is their geographical/ecological and phylogenetic relationship with the first batch of 60 strains.

10) The caption of Figure 3D can be extended to better clarify what the colour code of the bar indicates and better explain the heatmap on the top part of the figure.

11) Is it possible to add the histogram plot on Figure S7 so that the differences that are indicating in the figure caption can be easily spotted? Also, can the difference presented in the legend be tested more formally?

12) the caption of Figure S10 could be improved by adding some information regarding the heatmap S10A such as explaining the clustering of the strains, how the standardized value is calculated and add a clear calling of Figure S10B and C in the caption.

13) the following sentence could be rephrased for improving its clarity "Thus, knowing the individual effect of yeast strains can be useful to qualitatively infer their effect on the function of complex communities. However, exploring the simplest possible model -where the function of the community is calculated by the sum of the neutral or detrimental effect (dF) of each strain in the sugar consumption capacity of co-cultures with S. cerevisiae (Figure 3D)- we are far from quantitatively predicting the function of complex communities".

14) I am not entirely sure about the following sentence "The phylogenetic conservatism of microbial traits is required if we wish to predict the presence of different ecological functions in a community from its taxonomic composition". Here the authors are not trying to predicting the presence of ecological functions but their quantitative outcome.

15) this sentence "In this regard, we highlight some outstanding results, that may help, for instance, to contribute to mitigate the effects of climate change in the wine industry, such as the capacity of T. del-brueckii to grow in vitamins-deficient grape musts, or to modulate wine acidity by producing lactic acid (L. elongisporus and L. thermotolerans) or degrading malic (H. osmophila, L. elongisporus and P. kudriavzevii) or tartaric acid (C. amylolentus)." Lacks to point to where these results have been pre-sented (e.g. Figure XXX) because this is the first time in the manuscript these results are directly dis-cussed.
16) The following sentence seems to be in the wrong place in the discussion as this is part of the re-sults, and I would move it to the results section where the authors discuss the FEE application to pre-dicting community function "Additionally, given the success we had in using phylogenetic imputation to predict ecologically relevant traits in wine yeasts (Figure S4), we asked whether the FEE slope and intercept could also be interpreted as complex ecological traits, and thus, can be predicted in the same manner. Figure S10 shows a marginally significant correlation between observed and predicted FEEs parameters (R=0.54, for both slope and intercept), but it seems that the functional effect of yeast strains in complex communities cannot be clearly predicted by phylogeny".
17) The paragraph starting with "Moreover, we also looked for individual traits (among those de-scribed in Figure 2) that can be somehow related with the ecological effect of strains..." and finishing with "...we found that the ethanol production capacity, the consumption of organic nitrogen and the glucose:fructose consumption ratio, are important predictors of the effect that one strain may have to the function of wine yeast communities (Figure S11)" also seems misplaced in the discussion section and I think it would belong to the results section of the paper.
18) As presented in the Materials and methods section, it (incorrectly?) looks like the strains were all de novo isolated and identified for this work. If some strains isolated from previous works were used this should be mentioned here.

19) The way in which the FEE are concatenated to build the full model should be clarified better in the Materials and methods section.
20) The composition of the Sabouraud media should be reported in the materials and methods section.

Reviewer #2:

This is an interesting study that demonstrates the potential application of a microbiome engineering framework to industrial wine fermentation. The paper is made up of two 'chapters. In the first one, and to my mind minor chapter, the authors show that yeast function and phylogeny are positively correlated. In this case, the authors provide a compelling definition of function, by measuring the chemical composition of the wine fermentation medium where the organism has grown. The positive correlation

between phylogenetic distance and functional distance is consistent with the default expectation, since there must be some phylogenetic inertia on the pathways that mediate the relevant chemical transformations. However, the correlation does not seem very strong to me, which is perhaps explains why it does not help predict function in complex communities. Overall, although I do not find this first part of the paper very insightful, I do recognize it makes an important point.
The second, and to my mind more interesting 'chapter', adopts a quantitative framework previously described in a paper by the same senior author (Diaz-Colunga et al, 2022) to show how it can help one rationally design wine fermenting communities. Here, the authors assemble hundreds of low-complexity communities and try to predict the function of the community from the function of its constituents. The authors compare two models: in the first model the effects of individual taxa on community function (total fraction of sugars consumed) are simply added. In the second model, the effect of each taxon is assessed against the function of each of the background communities where is found, i.e. its community context, using the framework defined in Diaz-Colunga.

Major comments:
I would like to preface this section by saying that I feel quite positive about this paper, so my "major comments" are quite innocuous.
1. The additive model mentioned above is too naïve, and I interpret it as a strawman, because it's to be expected that the effects cannot be additive and must saturate - for example you cannot have negative consumption. This problem is inherited from the epistasis literature, where necessarily saturating fitness effects and true genetic interactions are treated the same way. To be clear, I do think it is important to mention that the linear model fails, but the authors need to add some nuance and perhaps motivate its use differently.
2. I find the Diaz-Colunga model quite compelling, but unfortunately it is poorly explained in the paper. In particular, the explanation of how the individual effects are combined is missing: In page 12, the function of the community is calculated by "concatenating the FEEs of each strain member of the community - as described by Diaz-Colunga et al. (2022)". What do you mean by "concatenating"? This needs to be explained in the text.
3. Overall, I would like to suggest the authors explain the framework of Diaz Colunga et al in more detail, rather than repeatedly citing this work. Perhaps a schematic would help. Then the paper could refer to the schematic multiple times, rather than to the citation.

Minor comment:
The paper is nicely written, but the quality of the language starts to decay in the discussion, especially towards the end.
Page 16, "the effect that one strain may have to the function .." should be "on the function".
Page 17, "we will address future works"
Page 17, "but, by now, we finish this work providing evidences"

Reviewer #3:

This manuscript examines the feasibility of predicting yeast community functions from the taxonomy of the constituent strains and species. The authors measured a panel of phenotypes for a collection of wine yeast strains, as well as hundreds of community combinations. They then integrated quantitative rules and linear models about the effect of strains on the phenotypes of the focal species S. cerevisiae, as well as communities, to predict phenotypes in communities of novel species combinations. I do not have the expertise to review the linear models or the FEEs. Apart from those, overall, this study describes a rational method to characterize microbial communities, and will be of interest to microbiologists, ecologists, and microbial biotechnology researchers. While most of their data supports their claims, I have a few concerns:

1) In Fig S4, the authors should clearly label or mark (perhaps in a different color?) the two traits that don't show a correlation with the phylogeny. How do the authors explain that the growth efficiency in malic acid correlated with the phylogeny, when they show in Fig 2D that its phylogenetic signal is low?

2) For Fig. 4A, the authors say that S. cerevisiae can completely consume sugars, and therefore show the dashed grey line, but in Figure 3C, they show that just S. cerevisiae alone (0 strains co-inoculated with S. cerevisiae) does not consume all sugars (appears to be between 90-95%). So perhaps they should show the dashed grey line accordingly - this would make it easier to visualize how the communities change the phenotype.

3) In Fig. 4B, what is the scale of the y-axis? Does it start from -1 ? The figures are very small, but Mp, Pk, and Hop appear to have very minor effects (dF) on S. cerevisiae containing communities (barring one or two exceptions) - the effect looks essentially neutral. This doesn't match the description of a "marked negative effect" (last lines on Page 11), or the data shown in Figure S7 and 3C. The authors should provide a larger figure, and perhaps a more quantitative description of the negative effects, to explain this discrepancy.

4) In Fig S3A, there appears to be a very low correlation between phenotypic distance and the wine physical-chemical parameters measured after 168h of growth in SGM. But the correlation of phenotypic distance to the growth efficiency in the 28 environmental preference assays is much higher (Fig. S3B). Instead of generalizing that there is high correlation for both (page

6), the authors can explicitly state the difference, and discuss what it may mean.

Minor comments:
1) In Figure 2A, the authors show the growth efficiencies of their strains in different environmental conditions. On Page 6, they say "In wine fermentation conditions, Ascomycetes yeasts exhibit, on average, higher growth efficiencies than basidiomycetes." However, for most of the basidiomycetes (light and dark green), the growth efficiency for 9/16 conditions seems higher than more than half of the ascomycetes (the ones represented by the orange squares, not purple). Perhaps the authors could either remove this statement, or make it more specific.

2) In Fig 3C, perhaps color code the replicates in which the species that have a negative impact on the growth efficiency of S. cerevisiae are present in the community? That way it would be easy to tell whether the low values tend to include those species?

3) In Fig S7, it is difficult to tell the differences between the different graphs, especially when they are not aligned below one another (e.g. is Ku different from Hop?). The authors should further annotate these graphs, e.g. by showing two vertical lines for all graphs that represent the 95% confidence intervals of S. cerevisiae sugar consumption. This would make it much easier to see the differences between graphs, and the differences between the phenotypes of the communities and S. cerevisiae alone.

4) Fig 2H : What does the inset show? Is the y-axis of the inset a ratio?

Reviewer #1:

Answer: We want to sincerely thank to the Reviewer #1 for their great efforts in reading and improving our work with their comments. We believe that our work is now better focused after addressing all the questions raised by the Reviewer #1. Our answer to the questions proposed by the reviewer can be found below.

This study aims at inferring microbial community (mixtures of wine yeast isolates) phenotypes related to wine fermentation by using a linear model (described in another publication) combining phylogenetic relatedness and individual species/strain phenotyping data. The phylogenetic relatedness is based on a single marker gene (26S rRNA) and phenotype defined using sugar consumption and growth/secretion parameters relevant for wine fermentation. The study uses 60 yeasts isolated from wine fermentation. While the overall concept is interesting and has possible biotechnological applications, there are several points where the study lacks evidence to support the conclusions drawn. There is neither concrete mechanistic insights into inter-strain interactions, nor any physiological insight into community-scale fermentation. Further, the genomic resolution is poor due to usage of single marker gene (as evident from Suppl Fig 1, where some of the isolates are identical). The conclusions drawn are thus not likely to be generalisable. Indeed, high-resolution studies in yeast (e.g., 1000 yeast project) have clearly shown that even genome sequencing is not sufficient to unravel complex genotype-phenotype relations in yeast.

Answer: We acknowledge Reviewer #1 comment regarding the lack of mechanistic explanations for the yeast interaction effects observed here. The goal of our study was primarily to quantify functional traits and interspecies interactions in yeast communities, and explore how these traits and interactions determine the function of a complex community. Indeed, numerous previous studies have already determined the mechanistic basis of pairwise interaction between wine yeast species, such as antimicrobial production or competition for nitrogen sources (we have now included more detailed information about these interaction mechanisms on page 17). A major novelty of our work is to illustrate that despite these complex interactions one can still predict the function of a complex community and relate those functions to strain-level traits. We have updated our introduction to more clearly state that our study focuses on identifying and quantifying the effects of these interactions rather than elucidating the underlying mechanisms. We plan to experimentally explore the mechanistic basis of the observed effects in the near future.

In addition, we do recognize that we have limited genomic resolution due to the use of a single gene marker to construct the phylogeny (which we have now acknowledged in the main text, page 15). In fact one of the striking results of our study is that despite the limited genomic resolution and presumed high interspecies diversity in many traits (as shown in the 1000 yeast project) the 26s gene is still able to provide information about many important functional traits as shown by the success of the phylogenetic imputation based prediction). In the new version of the manuscript we have further confirmed the robustness of these predictions by testing the imputation-based approach at different phylogenetic levels (see Figure S6 and comments). These results illustrate that even coarse grain genomic information from a single marker gene may be used to predict phenotypic differences when placed in a broad phylogenetic context. We acknowledge that additional markers would be needed to capture the variation at more narrow phylogenetic scales (such as amongst different strains from the same species and this is now stated in page

15). We also acknowledge that this approach is insufficient in predicting certain phenotypes (such as growth on malic acid as the sole carbon source), and that additional genomic resolution may be necessary for predicting traits with low phylogenetic signal (page 7).

The abstract, introduction, and discussion sections are overly generalized and also poorly contextualised (e.g., there is a single reference to 8 year old study for fermented food as model system and there is no explanation on in what way wine yeast community studies here makes a model - especially given no interaction network).

Answer: Thanks for your comment. We have reviewed these sections and have tried to avoid such general conclusions, replacing them with more specific statements about the scope of the work. Also, we have now included new references about the use of wine and other fermented foods in ecology studies (Conacher et al., 2022; Jahn et al., 2023), and justifying its use as a model system in ecology (page 4).

Below are some additional points that we hope will be useful.

1) The 60 yeast strains: Have these been obtained from the same fermentation environment? Can this be annotated? Following this, have the 53 strains that the authors phenotyped later in the first paragraph of the results been obtained from the same wine fermentations? By looking at the NCBI entries I understood that several of the strains were already isolated from previous works (e.g. Unraveling the Enzymatic Basis of Wine 'Flavorome': APhylo-Functional Study of Wine Related Yeast Species JOURNAL Front Microbiol 7, 12 (2016)) This information is vital because evolutionary processes such as population bottlenecks and adaptation to specific environmental niches, even different wine fermentations, as some of the authors point out in the mentioned publication above, can generate high phenotypic diversity in strains belonging to the same species. This would pose a challenge to inferring trait relatedness only using 26S as molecular marker though the signal may still be strong but it is not clear if that has been explored.

Answer: Thank you for this observation. The 60 strains come from a larger collection of wine yeasts, which were isolated from grape musts collected from different regions in Spain during different years, and this information has now been added to Table S1. We have also now annotated the phylogenetic tree in Figure S1 with the year and region from which the strains were isolated as the reviewer has suggested. Our collection includes some moderately related strains that were isolated from the same region (but different vineyards blocks) and year (such as Wa_3 and Wa_4) as well as some closely related strains isolated from different regions and years (such as Le_1 and Le_2). Disentangling the relative importance of different evolutionary processes in shaping phenotypic differences amongst close relatives is beyond the scope of this work, but we have now made note of this important point in the main text (pages 5 and 15).

2) branch length in the phylogenetic tree in figure S1?

Answer: According to the reviewer suggestion, the branch length scale is now added to Figure S1 (and Figure S7).

3) There are several mistakes in Table S1 at the annotation of the Family level. For instance, Brettanomyces isolates, Pichia isolates and Meyerozyma guillermondii isolates are annotated as family Saccharomycetacae but they do not belong to this family as the author showed in Figure 1B. Also, in legend of figure 1B is missing the explanation of the triangles at the end of the branches.

Answer: Thanks for the observation and apologies for the mistake. We have corrected the family names in Table S1, and now, the names match in table S1 and in Figure 2A and 2B. We have also included the family, order and division names of the isolates in Table S1. The triangles in Figure 1B refer to the different genera of the tree; the length of the triangle indicates the distance from the common ancestor of each strain included in the different genera (based on the phylogenetic tree shown on Figure S1). This point is now reflected in the footnote of Figure 1.

4) There is a mistake in the text when the authors report "Ascomycetes yeasts (purple and orange) exhibit, on average, higher growth efficiencies than basidiomycetes (light and dark green) (T-test, p=5.387e-07). Among Ascomycetes, Saccharomycetaceae strains (purple) have, on average, higher growth efficiencies than the non- Saccharomycetaceae strains (orange and light green) (T-test, p=1.604e-12)" Light green refers to Basidiomycetes according to the legend provided by the authors, so the second paragraph should not include the "light green" strains in the comparison as those are basidiomycetes. Also please revise the use of italics across the manuscript, in some passages is missing.

Answer: Thank you for noticing that. This mistake has been corrected but we have decided to remove the comparison of Ascomycetes versus Basidiomycetes growth efficiency (as Basidiomycetes group is underrepresented regarding the number of strains) and focus on the comparison between the growth of the Saccharomyceae group and all the other yeasts (Page 6).

We have also reviewed the use of italics throughout the manuscript.

5) In both Figure 2A and 2B the phylogenetic tree on the left is not aligned with the respective heatmap. Also, how the clustering using the phenotypic data looks like?

Answer: Corrected. Now the branches of the trees align correctly with the heatmap. As part of this correction, we have also updated the heatmap in figure 2B to include strain Bb_1 (Grey row in Figure 2B; this strain didn't grow in the microvinification assay reported in Fig. 2B, so it wasn’t included in the first version of this Figure, but we have now included it, as N/A data, for more clarity), and repeated all the analysis in this figure. Also, as suggested by the Reviewer, we have included as supplementary material the heatmaps representation by clustering based on the phenotypic data instead of the phylogeny (new Appendix Figure S4).

6) In figure S3 A the linear correlation is negligible R is less then 0.2 I would refrain to make strong affirmations given also the distribution of the points in the plot.

Answer: It is true that the correlation is weak in the case of fermentation performance data. We have now moderated the claims throughout the manuscript and specified that the correlation regarding environmental preferences phenotype is stronger, and also, we hypothesize about the causes of these differences found (see pages 6, 14). We acknowledge that, in the context of fermentative performance assays, there may be unmeasured variables present in the experimental conditions that can interact with and impact the concentration of metabolites and physicochemical parameters. This can result in a reduced correlation between the phenotype and the phylogeny, ultimately making the outcomes less predictable.

7) In the legend of Figure 2A "...indicates that the trait distribution amongst strains is highly influenced by their shared evolutionary history." I would avoid using the term "shared" with "evolutionary histories" in this context and maintain only "evolutionary history". The correlation implies that the phenotypic distance and the phylogenetic distance have a non-random relationship but it does not imply that the strains have "shared" evolutionary histories.

Answer: Thank you for the suggestion. The sentence has been rewritten accordingly (legend of Figure 2A, page 38).

8) In Figure 2E the authors used the "Leave one out cross validation (LOOCV)" to validate their phenotypic imputation based on the phylogeny. I wondered if performing the same analysis by leaving out all but one strains from each phylogenetic unit at the species level would decrease the robustness of the correlation of the prediction versus the measured values. As phylopar re-roots to the closer common ancestor there are many cases where the LOOCV would use the sister strains from the same species with very low phylogenetic distance for re-rooting and impute a phenotype. This aspect is also pointed out by the authors "Predictions are also better at shorter phylogenetic distances, when there are closer relatives present in the tree" and I think it could be worth exploring it to assess the limit of the approach.

Answer: Thank you for this comment. Inspired by your remarks we have rerun this analysis at different taxonomic levels. For each of the 60 strains we repeated the phylogenetic imputation of phenotypic traits using a) all other strains (as in the main text and original submission) b) all strains with different 26s sequence c) all strains belonging to a different species and d) all strains belonging to different genera. For each trait we calculated the correlation between predicted and observed trait values using different amounts of taxonomic information (new Appendix Figure S6). As expected, the correlation between predicted and observed trait values decreases as closely related strains are removed. However, even in the worst case scenario, where trait values are imputed without using information from any strains in the same genus, we find that 22/43 traits show a significant positive correlation between predicted and observed trait values.

9) Referring to point 1) It would help to clarify where the 53 strains have been obtained and what is their geographical/ecological and phylogenetic relationship with the first batch of 60 strains.

Answer: The 53 strains come from the same larger collection of wine yeasts strains. The additional information of these strains is now included in Table S5. We also now included a phylogenetic tree including all 113 strains (the 53 new strains and the first batch of 60 strains) and annotated this tree with the geographical origin and year of isolation (Figure S7).

10) The caption of Figure 3D can be extended to better clarify what the colour code of the bar indicates and better explain the heatmap on the top part of the figure.

Answer: According to the reviewer's suggestion, we have increased the size of the caption and changed the color patterns to make easier the appreciation of differences in dF of the strains with respect to *S. cerevisiae* function (sugar consumption). Additionally, we have included scale values and a dF label to provide a better explanation of the heatmap (Figure 3D).

11) Is it possible to add the histogram plot on Figure S7 so that the differences that are indicating in the figure caption can be easily spotted? Also, can the difference presented in the legend be tested more formally?

Answer: according to the reviewer's suggestion, we have tried showing the histograms, but the differences were not clearly appreciated. According to reviewer 3 suggestion, we have included vertical lines that make it easier to compare the curves from different strains.

In addition, the strains are ordered on the panels based on the percentage of the communities that consumed more than the 90% of the sugars. This annotation is now included in the footnote of the figure (now Figure S11).

Also, we performed an ANOVA test to examine the differences in the distribution of strains across the different communities assayed, basing on the sugar consumption function. Specifically, we compared the strains based on their presence in high-functioning communities, i.e., communities that consume over 90% of the sugars. The results of this test are now shown in Figure S11, and the test explained in the figure note.

12) the caption of Figure S10 could be improved by adding some information regarding the heatmap S10A such as explaining the clustering of the strains, how the standardized value is calculated and add a clear calling of Figure S10B and C in the caption.

Answer: The caption of the figure (now Figure S15) has been improved according to the reviewer's suggestion.

13) the following sentence could be rephrased for improving its clarity "Thus, knowing the individual effect of yeast strains can be useful to qualitatively infer their effect on the function of complex communities. However, exploring the simplest possible model -where the function of the community is calculated by the sum of the neutral or detrimental effect (dF) of each strain in the sugar consumption capacity of co-cultures with S. cerevisiae

(Figure 3D)- we are far from quantitatively predicting the function of complex communities".

Answer: The sentence has been rewritten to improve its clarity (Page 10).

14) I am not entirely sure about the following sentence "The phylogenetic conservatism of microbial traits is required if we wish to predict the presence of different ecological functions in a community from its taxonomic composition". Here the authors are not trying to predicting the presence of ecological functions but their quantitative outcome.

Answer: The sentence has been corrected according to the reviewer's suggestion (Page 13).

15) this sentence "In this regard, we highlight some outstanding results, that may help, for instance, to contribute to mitigate the effects of climate change in the wine industry, such as the capacity of T. delbrueckii to grow in vitamins-deficient grape musts, or to modulate wine acidity by producing lactic acid (L. elongisporus and L. thermotolerans) or degrading malic (H. osmophila, L. elongisporus and P. kudriavzevii) or tartaric acid (C. amylolentus)." Lacks to point to where these results have been pre-sented (e.g. Figure XXX) because this is the first time in the manuscript these results are directly dis-cussed.

Answer: Thank you for noticing that. We have added the references to Figures 2A and 2B in this sentence (Page 16).

16) The following sentence seems to be in the wrong place in the discussion as this is part of the re-sults, and I would move it to the results section where the authors discuss the FEE application to pre-dicting community function "Additionally, given the success we had in using phylogenetic imputation to predict ecologically relevant traits in wine yeasts (Figure S4), we asked whether the FEE slope and intercept could also be interpreted as complex ecological traits, and thus, can be predicted in the same manner. Figure S10 shows a marginally significant correlation between observed and predicted FEEs parameters (R=0.54, for both slope and intercept), but it seems that the functional effect of yeast strains in complex communities cannot be clearly predicted by phylogeny".

Answer: We have moved this paragraph to the results section according to the reviewer´s suggestion (now on page 12).

17) The paragraph starting with "Moreover, we also looked for individual traits (among those de-scribed in Figure 2) that can be somehow related with the ecological effect of strains..." and finishing with "...we found that the ethanol production capacity, the consumption of organic nitrogen and the glucose:fructose consumption ratio, are important predictors of the effect that one strain may have to the function of wine yeast communities (Figure S11)" also seems misplaced in the discussion section and I think it would belong to the results section of the paper.

Answer: This paragraph is now placed in the results section (now on page 13).

18) As presented in the Materials and methods section, it (incorrectly?) looks like the strains were all de novo isolated and identified for this work. If some strains isolated from previous works were used this should be mentioned here.

Answer: As mentioned before, according to the reviewer's suggestion, new information about the origin of the strains is now included in Table S1 and shown on Appendix Figure S1.

19) The way in which the FEE are concatenated to build the full model should be clarified better in the Materials and methods section.

Answer: According to the reviewer's suggestion, we have rewrite this section, clarifying the procedure followed to calculate the FEEs and to integrate them in the model (Pages 28 and 29).

20) The composition of the Sabouraud media should be reported in the materials and methods section.

Answer: added according to the reviewer's suggestion (page 19).

Reviewer #2:

This is an interesting study that demonstrates the potential application of a microbiome engineering framework to industrial wine fermentation. The paper is made up of two 'chapters. In the first one, and to my mind minor chapter, the authors show that yeast function and phylogeny are positively correlated. In this case, the authors provide a compelling definition of function, by measuring the chemical composition of the wine fermentation medium where the organism has grown. The positive correlation between phylogenetic distance and functional distance is consistent with the default expectation, since there must be some phylogenetic inertia on the pathways that mediate the relevant chemical transformations. However, the correlation does not seem very strong to me, which is perhaps explains why it does not help predict function in complex communities. Overall, although I do not find this first part of the paper very insightful, I do recognize it makes an important point.
The second, and to my mind more interesting 'chapter', adopts a quantitative framework previously described in a paper by the same senior author (Diaz-Colunga et al, 2022) to show how it can help one rationally design wine fermenting communities. Here, the authors assemble hundreds of low-complexity communities and try to predict the function of the community from the function of its constituents. The authors compare two models: in the first model the effects of individual taxa on community function (total fraction of sugars consumed) are simply added. In the second model, the effect of each taxon is assessed against the function of each of the background communities where is found, i.e. its community context, using the framework defined in Diaz-Colunga.
Major comments:
I would like to preface this section by saying that I feel quite positive about this paper, so my "major comments" are quite innocuous.

Answer: Thanks to Reviewer #2 for their kind words and the efforts in reviewing our work. We think their suggestions have substantially improved the manuscript.

1. The additive model mentioned above is too naïve, and I interpret it as a strawman, because it's to be expected that the effects cannot be additive and must saturate - for

example you cannot have negative consumption. This problem is inherited from the epistasis literature, where necessarily saturating fitness effects and true genetic interactions are treated the same way. To be clear, I do think it is important to mention that the linear model fails, but the authors need to add some nuance and perhaps motivate its use differently.

Answer: Thank you for this observation. We totally agree with your comment and we recognize that the additive model may be a kind of strawman. Also, it is worth mentioning that the additive model presented here is not based on the sum of the functional effect of the strains on the community function but subtracts the functional effects of each species in co-culture with *S. cerevisiae* precisely to avoid saturation. We have now clarified this in the main text and mentioned that the additive model was used to test for the existence of higher-order interactions in our system, not with the expectation that it would actually work (pages 10 and 27).

2. I find the Diaz-Colunga model quite compelling, but unfortunately it is poorly explained in the paper. In particular, the explanation of how the individual effects are combined is missing: In page 12, the function of the community is calculated by "concatenating the FEEs of each strain member of the community - as described by Diaz-Colunga et al. (2022)". What do you mean by "concatenating"? This needs to be explained in the text.

Answer: Thanks to this comment and according to the suggestions of other reviewers, the new version of the manuscript has a better and more complete explanation about how the FEEs model described by Diaz-Colunga et al. (2022) has been applied here (pages 28 and 29).

3. Overall, I would like to suggest the authors explain the framework of Diaz Colunga et al in more detail, rather than repeatedly citing this work. Perhaps a schematic would help. Then the paper could refer to the schematic multiple times, rather than to the citation.

Answer: Thank you for your suggestion. We have prepared a new and more detailed paragraph with a better explanation of the methodology followed (pages 28 and 29), so the work now has greater autonomy, and we can relieve the text of so many cross references to Diaz-Colunga's paper.

Minor comment:
The paper is nicely written, but the quality of the language starts to decay in the discussion, especially towards the end.

Answer: Thanks for noticing, we have overall revised the writing style and the use of English in the manuscript, especially in that section.

Page 16, "the effect that one strain may have to the function .." should be "on the function".

Answer: changed according to the reviewer’s suggestion (Page 13)

Page 17, "we will address future works"

Answer: changed according to the reviewer’s suggestion (Page 13)

Page 17, "but, by now, we finish this work providing evidences"

Answer: changed according to the reviewer’s suggestion (Page 13)

Reviewer #3:

This manuscript examines the feasibility of predicting yeast community functions from the taxonomy of the constituent strains and species. The authors measured a panel of phenotypes for a collection of wine yeast strains, as well as hundreds of community combinations. They then integrated quantitative rules and linear models about the effect of strains on the phenotypes of the focal species S. cerevisiae, as well as communities, to predict phenotypes in communities of novel species combinations. I do not have the expertise to review the linear models or the FEEs. Apart from those, overall, this study describes a rational method to characterize microbial communities, and will be of interest to microbiologists, ecologists, and microbial biotechnology researchers. While most of their data supports their claims, I have a few concerns:

Answer: We would like to thank to Reviewer #3 for their work and effort in reviewing our manuscript and for the useful comments raised that are now considered in this new version of the work.

1) In Fig S4, the authors should clearly label or mark (perhaps in a different color?) the two traits that don't show a correlation with the phylogeny. How do the authors explain that the growth efficiency in malic acid correlated with the phylogeny, when they show in Fig 2D that its phylogenetic signal is low?

Answer: Thank you for this suggestion. We have changed the Fig S4 (now Fig. S5) by ordering the traits based on the correlation value, and also marked in red those traits that do not exhibit significant correlation with the phylogeny.

We want to thank the reviewer for this comment which led us to more carefully interrogate the significance of the phylogenetic signal calculation shown in figure 2D. Phytools calculates the p.value for Pagels λ using a likelihood ratio test which assumes a chi-square distribution of this statistic (see Munkelmuller et al 2012 for more details). As noted in this paper this assumption is simply for the purposes of computational efficiency (for very large phylogenies calculating lambda can be time consuming). The more appropriate statistical test would be to compare the observed estimate of λ with the distribution obtained when randomly permuting the trait values among the tips of the phylogenetic tree. We have therefore repeated our analysis using this permutation test (with 1000 iterations) and have updated our manuscript and results accordingly. Using this permutation test we find that all traits exhibit a significant phylogenetic signal (Figure 2D) though the estimate is unchanged. This helps to explain why a positive correlation is observed between predicted and observed trait values for all traits in Figure 2D. Furthermore we find that growth efficiency on malic acid displays the lowest phylogenetic signals and concurrently has the lowest correlation between predicted and observed trait value (noted on page 7).

2) For Fig. 4A, the authors say that S. cerevisiae can completely consume sugars, and therefore show the dashed grey line, but in Figure 3C, they show that just S. cerevisiae alone (0 strains co-inoculated with S. cerevisiae) does not consume all sugars (appears to

be between 90-95%). So perhaps they should show the dashed grey line accordingly - this would make it easier to visualize how the communities change the phenotype.

Answer: Thank you for this suggestion. You are right, and actually, in our experimental conditions, *S. cerevisiae* consumes on average 94.33% of the sugars when it is inoculated alone, which can be considered as a finished alcoholic wine fermentation in industrial terms.. We have now indicated that *S. cerevisiae* is able to complete wine fermentation as it is able to deplete around 95% of the fermentable sugars, avoiding to say: "can completely consume all the sugars". In addition, we have redo the Figure 4A by showing two dashed lines indicating the consumption of the 100% and the 95% of the sugars, respectively, representing the theoretical and empirical situation of *S. cerevisiae* function when it is inoculated in monoculture.

3) In Fig. 4B, what is the scale of the y-axis? Does it start from -1 ? The figures are very small, but Mp, Pk, and Hop appear to have very minor effects (dF) on S. cerevisiae containing communities (barring one or two exceptions) - the effect looks essentially neutral. This doesn't match the description of a "marked negative effect" (last lines on Page 11), or the data shown in Figure S7 and 3C. The authors should provide a larger figure, and perhaps a more quantitative description of the negative effects, to explain this discrepancy.

Answer: According to the reviewer suggestion, we have prepared a bigger version of Figure 4B. In addition, we have included a new supplementary figure where we show the average functional effect of each strain on the different background communities assayed (Figure S12). We think it helps to better discuss the functional effect of the strains, identifying those strains that consistently exert a detrimental impact on community function.

4) In Fig S3A, there appears to be a very low correlation between phenotypic distance and the wine physical-chemical parameters measured after 168h of growth in SGM. But the correlation of phenotypic distance to the growth efficiency in the 28 environmental preference assays is much higher (Fig. S3B). Instead of generalizing that there is high correlation for both (page 6), the authors can explicitly state the difference, and discuss what it may mean.

Answer: Thank you for the comment; we totally agree. We have now specified that the correlation is much stronger in the case of environmental preferences than in the case of fermentation performance throughout the manuscript, avoiding generalizing.

Now, we argue in the main text that, although the production of the metabolites that determines the wine physical-chemical parameters did not exhibit strong correlation between the phylogenetic and the phenotypic distance, the phylogenetic signals estimate are strong enough to predict most of this traits based on the phylogeny.

Also, it is worth mentioning that, compared to the assays for environmental preferences, where the measured variable was the change in optical density at 60 hours in the culture, the fermentative performance assays measure the final concentration of specific metabolites or certain physicochemical parameters at 7 days. Due to the inherent experimental nature of the latter, there are some non-controllable variables that could

interact and affect the concentration of metabolites and the physicochemical parameters, making the measurement less correlated with the phylogeny and, therefore, less predictable.

These arguments are now included in the main text (Pages 6 and 14).

Minor comments:
1) In Figure 2A, the authors show the growth efficiencies of their strains in different environmental conditions. On Page 6, they say "In wine fermentation conditions, Ascomycetes yeasts exhibit, on average, higher growth efficiencies than basidiomycetes." However, for most of the basidiomycetes (light and dark green), the growth efficiency for 9/16 conditions seems higher than more than half of the ascomycetes (the ones represented by the orange squares, not purple). Perhaps the authors could either remove this statement, or make it more specific.

Answer: Thank you for noticing that. In the first version of the manuscript, we made a mistake by including the light green strains in the group of Basidiomycetes. However, these strains are actually Ascomycetes (i.e. *Schizosaccharomyces* and *Aureobasidium* genera). In our collection, the Basidiomycetes are limited to *Cryptococcus*, *Naganishia*, and *Rhodotorula* strains (dark green). However, based on the suggestions from other reviewers, we have removed the comparison of Ascomycetes versus Basidiomycetes growth efficiency and focus on the comparison between the growth of the Saccharomyceae group and all the other yeasts, in order to support the central role of *S. cerevisiae* in our study (page 6).

2) In Fig 3C, perhaps color code the replicates in which the species that have a negative impact on the growth efficiency of S. cerevisiae are present in the community? That way it would be easy to tell whether the low values tend to include those species?

Answer: Thank you for the suggestion. We have created a new supplementary figure (Appendix Figure S10) that represents how the strains are distributed amongst the communities represented in the Figure 3C, making it easier to identify those that tend to be found in those communities with a lower function. This, together with the new plot showed in Appendix Figure S12 -that represents the change in function (ΔF value) that every strain cause on the community where they are inoculated-, makes it easier to identify those species causing a loss of function in the community.

3) In Fig S7, it is difficult to tell the differences between the different graphs, especially when they are not aligned below one another (e.g. is Ku different from Hop?). The authors should further annotate these graphs, e.g. by showing two vertical lines for all graphs that represent the 95% confidence intervals of S. cerevisiae sugar consumption. This would make it much easier to see the differences between graphs, and the differences between the phenotypes of the communities and S. cerevisiae alone.

Answer: Thank you for this suggestion. We have included vertical lines that make it easier to compare the curves from different strains and we think that the figure is now clearer. Also, the plots are ordered based on the number of *S. cerevisiae*-containing communities that consumed more than 90% of the sugars, and an ANOVA test was performed to identify the existence of statistical differences in the strains based on their presence in

high-functioning communities, i.e., communities that consume at least the 90% of the sugars. This information is now included in the footnote of Figure S11.

4) Fig 2H: What does the inset show? Is the y-axis of the inset a ratio?

Answer: We have rephrased the footnote referring to figure 2H in order to clarify the meaning of the inset figure. This figure shows how prediction accuracy for individual strains (i.e. difference between predicted and observed trait values) relates with the distance of the strain in the tree to its nearest neighbour (measured as the phylogenetic nearest neighbor distance, PNND).

24th Jul 2023

Manuscript Number: MSB-2023-11613R
Title: Predictability of the community-function landscape in wine yeast ecosystems

Dear Dr Belda,

Thank you for sending us your revised manuscript. We have now heard back from the three reviewers who were asked to evaluate your revised study. As you will see below, the reviewers are satisfied with the performed revisions and support publication. Reviewer #1 is still concerned about the lack of physiological and mechanistic insight. However, as my colleague Jingyi Hou had indicated in her initial decision letter, we think that addressing these issues by additional discussion and by contextualizing the related claims, seems sufficient. As such, we do not think further revisions are required regarding these points. Reviewer #3 lists a couple of minor concerns which we would ask you to address in a minor revision.

We would also ask you to address some remaining editorial issues listed below.

- Our data editors have noticed some unclear or missing information in the figure legends, please see the attached .doc file. Please make all requested text changes using the attached file and *keeping the "track changes" mode* so that we can easily access the edits made.

- The keywords need to be reduced to 5.

- Please include callouts to Appendix Figure S3A-B, S4A-B, S6A-D, S15A-C, S16A-D.

- The EV Tables (or Datasets) should be uploaded individually (ie. one file per EV Table of EV Dataset). Each file should include a description of the table/dataset in a separate sheet in the Excel file. Tables EV2-EV3 should be renamed to Tables EV1-EV2.

- Table EV1, EV4-EV8 should be uploaded as EV Datasets (Datasets EV1-EV6) as they are rather long. Please make sure that the callouts are updated accordingly.

- Pease include page numbers in the Appendix Table of Contents.

- Source Data: Please provide one file (or .zip folder) per main figure and a single .zip folder for all EV and/or Appendix figure Source Data.

Please resubmit your revised manuscript online, with a covering letter listing amendments and responses to each point raised by the referees. Please resubmit the paper **within one month** and ideally as soon as possible. If we do not receive the revised manuscript within this time period, the file might be closed and any subsequent resubmission would be treated as a new manuscript. Please use the Manuscript Number (above) in all correspondence.

Click on the link below to submit your revised paper.

https://msb.msubmit.net/cgi-bin/main.plex

As a matter of course, please make sure that you have correctly followed the instructions for authors as given on the submission website.

Thank you for submitting this paper to Molecular Systems Biology.

Kind regards,

Maria

Maria Polychronidou, PhD
Senior Editor
Molecular Systems Biology

If you do choose to resubmit, please click on the link below to submit the revision online before 23rd Aug 2023.

https://msb.msubmit.net/cgi-bin/main.plex

IMPORTANT:

Please note that corresponding authors are required to supply an ORCID ID for their name upon submission of a revised manuscript (EMBO Press signed a joint statement to encourage ORCID adoption).
(https://www.embopress.org/page/journal/17444292/authorguide#editorialprocess)
Currently, our records indicate that the ORCID for your account is 0000-0002-2607-5049.

Please click the link below to modify this ORCID:
Link Not Available

The system will prompt you to fill in your funding and payment information. This will allow Wiley to send you a quote for the article processing charge (APC) in case of acceptance. This quote takes into account any reduction or fee waivers that you may be eligible for. Authors do not need to pay any fees before their manuscript is accepted and transferred to the publisher.

*** PLEASE NOTE *** As part of the EMBO Press transparent editorial process initiative (see our Editorial at https://dx.doi.org/10.1038/msb.2010.72 , Molecular Systems Biology will publish online a Review Process File to accompany accepted manuscripts. When preparing your letter of response, please be aware that in the event of acceptance, your cover letter/point-by-point document will be included as part of this File, which will be available to the scientific community. More information about this initiative is available in our Instructions to Authors. If you have any questions about this initiative, please contact the editorial office (msb@embo.org).

Reviewer #1:

While I recognise efforts by the authors to improve the clarity, my original impression that "There is neither concrete mechanistic insights into inter-strain interactions, nor any physiological insight into community-scale fermentation." remains as no additional data is presented to directly address these.

Reviewer #2:

After carefully re-reading the revised manuscript and rebuttal letter, I have not further concerns. The authors have done a great job and I look forward to seeing this paper published.

Reviewer #3:

This is a revision of a manuscript on predicting yeast community wine-fermentation functions basedon the taxonomy of constituent strains and species. The authors have addressed all concerns, and I have no further questions. One minor comment is that some of the writing is unclear, and there are some typos in the text, so these should be clarified. A couple of examples are:
- Last paragraph of results should be "for now..." instead of "by now..."
- In the Discussion section, on Page 15, it is not clear what this sentence means "Thus, we stand out the relevance of the dataset and predictive tools provided in this work to easily predict wine-relevant traits of new taxa, only requiring their phylogenetic information provided by a single gene marker (26S sequence)." Do the authors mean "Thus, the relevance of the dataset and predictive tools provided in this work easily predict wine-relevant traits of new taxa, only requiring their phylogenetic information provided by a single gene marker (26S sequence)" ?

Detailed response to minor revisions requested

Manuscript Number: MSB-2023-11613R
Title: Predictability of the community-function landscape in wine yeast ecosystems

Comments from the editor

Dear Dr Belda,

Thank you for sending us your revised manuscript. We have now heard back from the three reviewers who were asked to evaluate your revised study. As you will see below, the reviewers are satisfied with the performed revisions and support publication. Reviewer #1 is still concerned about the lack of physiological and mechanistic insight. However, as my colleague Jingyi Hou had indicated in her initial decision letter, we think that addressing these issues by additional discussion and by contextualizing the related claims, seems sufficient. As such, we do not think further revisions are required regarding these points. Reviewer #3 lists a couple of minor concerns which we would ask you to address in a minor revision.

We would also ask you to address some remaining editorial issues listed below.

- Our data editors have noticed some unclear or missing information in the figure legends, please see the attached .doc file. Please make all requested text changes using the attached file and *keeping the "track changes" mode* so that we can easily access the edits made.

Response: All the changes proposed have been addressed in the manuscript file (keeping the track changes mode).

- The keywords need to be reduced to 5.

Response: The number of keywords has been now reduced to 5 (Microbial interactions, Community-Function landscape, Wine yeasts, Phylogenetic signal, Functional Effect Equations).

- Please include callouts to Appendix Figure S3A-B, S4A-B, S6A-D, S15A-C, S16A-D.

Response: All Appendix Figures (including all figure panels) are now mentioned in the text.

- The EV Tables (or Datasets) should be uploaded individually (ie. one file per EV Table of EV Dataset). Each file should include a description of the table/dataset in a separate sheet in the Excel file. Tables EV2-EV3 should be renamed to Tables EV1-EV2.

Response: We have created an individual file for each supplementary EV table (including the legend in an additional Excel sheet).

- Table EV1, EV4-EV8 should be uploaded as EV Datasets (Datasets EV1-EV6) as they are rather long. Please make sure that the callouts are updated accordingly.

Response: We have converted these tables to EV Datasets. Accordingly, the tables and datasets have been renamed in the manuscript, ensuring that all references in the text are correct.

- Pease include page numbers in the Appendix Table of Contents.

Response: We have included the page numbers in the Appendix file, indicating the specific number for each Appendix figure.

- Source Data: Please provide one file (or .zip folder) per main figure and a single .zip folder for all EV and/or Appendix figure Source Data.

Response: We have prepared an individual zip folder for the data sources per each main figure.

Comments from the reviewers

Reviewer #1:

While I recognise efforts by the authors to improve the clarity, my original impression that "There is neither concrete mechanistic insights into inter-strain interactions, nor any physiological insight into community-scale fermentation." remains as no additional data is presented to directly address these.

Answer: We would like to thank the reviewer #1 for their valuable comments and dedication throughout the revision process. We acknowledge that our work still lacks a comprehensive physiological and mechanistic explanation of the yeast interaction phenomena described. However, we want to emphasize that our primary objective was to quantify functional traits and interspecies interactions within yeast communities, aiming to understand how these factors shape the overall function of a complex community. Thus, as noted by the editor, the modifications made in the latest revision have provided new discussions and contextualization of the claims exposed in the manuscript.

Reviewer #2:

After carefully re-reading the revised manuscript and rebuttal letter, I have not further concerns. The authors have done a great job and I look forward to seeing this paper published.

Answer: We want to really thank the nice words of reviewer #2 and all her/his effort during the revision.

Reviewer #3:

This is a revision of a manuscript on predicting yeast community wine-fermentation functions based on the taxonomy of constituent strains and species. The authors have addressed all concerns, and I have no further questions.

Answer: We want to thank again the effort of reviewer #3 that help us to improve the quality of our work.

One minor comment is that some of the writing is unclear, and there are some typos in the text, so these should be clarified.

Answer: Thank you for the comment. We have reviewed the writing throughout the manuscript to avoid these typos and unclear sentences.

A couple of examples are:

- Last paragraph of results should be "for now..." instead of "by now..."

Answer: Corrected.

- In the Discussion section, on Page 15, it is not clear what this sentence means "Thus, we stand out the relevance of the dataset and predictive tools provided in this work to easily predict wine-relevant traits of new taxa, only requiring their phylogenetic information provided by a single gene marker (26S sequence)." Do the authors mean "Thus, the relevance of the dataset and predictive tools provided in this work easily predict wine-relevant traits of new taxa, only requiring their phylogenetic information provided by a single gene marker (26S sequence)"?

Answer: Thank you for notice that, we have clarified the sentence to make it more comprehensible.

26th Jul 2023

Manuscript number: MSB-2023-11613RR
Title: Predictability of the community-function landscape in wine yeast ecosystems

Dear Dr Belda,

Thank you again for sending us your revised manuscript. We are now satisfied with the modifications made and I am pleased to inform you that your paper has been accepted for publication.

*** PLEASE NOTE *** As part of the EMBO Publications transparent editorial process initiative (see our Editorial at https://dx.doi.org/10.1038/msb.2010.72), Molecular Systems Biology publishes online a Review Process File with each accepted manuscripts. This file will be published in conjunction with your paper and will include the anonymous referee reports, your point- by-point response and all pertinent correspondence relating to the manuscript. If you do NOT want this File to be published, please inform the editorial office at msb@embo.org within 14 days upon receipt of the present letter.

Should you be planning a Press Release on your article, please get in contact with msb@wiley.com as early as possible, in order to coordinate publication and release dates.

LICENSE AND PAYMENT:
All articles published in Molecular Systems Biology are fully open access: immediately and freely available to read, download and share.

Molecular Systems Biology charges an article processing charge (APC) to cover the publication costs. You, as the corresponding author for this manuscript, should have already received a quote with the article processing fee separately. Please let us know in case this quote has not been received.

Once your article is at Wiley for editorial production you will receive an email from Wiley's Author Services system, which will ask you to log in and will present you with the publication license form for completion. Within the same system the publication fee can be paid by credit card, an invoice or pro forma can be requested.

Payment of the publication charge and the signed Open Access Agreement form must be received before the article can be published online.

Molecular Systems Biology articles are published under the Creative Commons licence CC BY, which facilitates the sharing of scientific information by reducing legal barriers, while mandating attribution of the source in accordance to standard scholarly practice.

Proofs will be forwarded to you within the next 2-3 weeks.

Thank you very much for submitting your work to Molecular Systems Biology.

Sincerely,

Maria Polychronidou, PhD
Senior Editor
Molecular Systems Biology

EMBO Press Author Checklist

Corresponding Author Name: Ignacio Belda	**USEFUL LINKS FOR COMPLETING THIS FORM**
Journal Submitted to: Molecular Systems Biology	The EMBO Journal - Author Guidelines
Manuscript Number: MSB-2023-11613	EMBO Reports - Author Guidelines
	Molecular Systems Biology - Author Guidelines
	EMBO Molecular Medicine - Author Guidelines

Reporting Checklist for Life Science Articles (updated January 2022)
This checklist is adapted from Materials Design Analysis Reporting (MDAR) Checklist for Authors. MDAR establishes a minimum set of requirements in transparent reporting in the life sciences (see Statement of Task: 10.31222/osf.io/9sm4x). Please follow the journal's guidelines in preparing your manuscript.
Please note that a copy of this checklist will be published alongside your article.

Abridged guidelines for figures
1. Data
The data shown in figures should satisfy the following conditions:
- the data were obtained and processed according to the field's best practice and are presented to reflect the results of the experiments in an accurate and unbiased manner.
- ideally, figure panels should include only measurements that are directly comparable to each other and obtained with the same assay.
- plots include clearly labeled error bars for independent experiments and sample sizes. Unless justified, error bars should not be shown for technical replicates.
- if n<5, the individual data points from each experiment should be plotted. Any statistical test employed should be justified.
- Source Data should be included to report the data underlying figures according to the guidelines set out in the authorship guidelines on Data Presentation.

2. Captions
Each figure caption should contain the following information, for each panel where they are relevant:
- a specification of the experimental system investigated (eg cell line, species name).
- the assay(s) and method(s) used to carry out the reported observations and measurements.
- an explicit mention of the biological and chemical entity(ies) that are being measured.
- an explicit mention of the biological and chemical entity(ies) that are altered/varied/perturbed in a controlled manner.
- the exact sample size (n) for each experimental group/condition, given as a number, not a range;
- a description of the sample collection allowing the reader to understand whether the samples represent technical or biological replicates (including how many animals, litters, cultures, etc.).
- a statement of how many times the experiment shown was independently replicated in the laboratory.
- definitions of statistical methods and measures:
  - common tests, such as t-test (please specify whether paired vs. unpaired), simple χ2 tests, Wilcoxon and Mann-Whitney tests, can be unambiguously identified by name only, but more complex techniques should be described in the methods section;
  - are tests one-sided or two-sided?
  - are there adjustments for multiple comparisons?
  - exact statistical test results, e.g., P values = x but not P values < x;
  - definition of 'center values' as median or average;
  - definition of error bars as s.d. or s.e.m.

Please complete ALL of the questions below.
Select "Not Applicable" only when the requested information is not relevant for your study.

Materials

Newly Created Materials	Information included in the manuscript?	In which section is the information available? (Reagents and Tools Table, Materials and Methods, Figures, Data Availability Section)
New materials and reagents need to be available; do any restrictions apply?	Not Applicable	

Antibodies	Information included in the manuscript?	In which section is the information available? (Reagents and Tools Table, Materials and Methods, Figures, Data Availability Section)
For **antibodies** provide the following information:<br>- Commercial antibodies: RRID (if possible) or supplier name, catalogue number and or/clone number<br>- Non-commercial: RRID or citation	Not Applicable	

DNA and RNA sequences	Information included in the manuscript?	In which section is the information available? (Reagents and Tools Table, Materials and Methods, Figures, Data Availability Section)
**Short novel DNA or RNA including primers, probes**: provide the sequences.	Not Applicable	

Cell materials	Information included in the manuscript?	In which section is the information available? (Reagents and Tools Table, Materials and Methods, Figures, Data Availability Section)
**Cell lines:** Provide species information, strain. Provide accession number in repository **OR** supplier name, catalog number, clone number, and/**OR** RRID.	Not Applicable	
**Primary cultures:** Provide species, strain, sex of origin, genetic modification status.	Not Applicable	
Report if the cell lines were recently **authenticated** (e.g., by STR profiling) and tested for mycoplasma contamination.	Not Applicable	

Experimental animals	Information included in the manuscript?	In which section is the information available? (Reagents and Tools Table, Materials and Methods, Figures, Data Availability Section)
**Laboratory animals or Model organisms:** Provide species, strain, sex, age, genetic modification status. Provide accession number in repository **OR** supplier name, catalog number, clone number, **OR** RRID.	Not Applicable	
**Animal observed in or captured from the field:** Provide species, sex, and age where possible.	Not Applicable	
Please detail **housing and husbandry conditions**.	Not Applicable	

Plants and microbes	Information included in the manuscript?	In which section is the information available? (Reagents and Tools Table, Materials and Methods, Figures, Data Availability Section)
**Plants:** provide species and strain, ecotype and cultivar where relevant, unique accession number if available, and source (including location for collected wild specimens).	Not Applicable	
**Microbes:** provide species and strain, unique accession number if available, and source.	Yes	Material and Mehods and Data Availability Section

Human research participants	Information included in the manuscript?	In which section is the information available? (Reagents and Tools Table, Materials and Methods, Figures, Data Availability Section)
If collected and within the bounds of privacy constraints report on age, sex and gender or ethnicity for all study participants.	Not Applicable	

Core facilities	Information included in the manuscript?	In which section is the information available? (Reagents and Tools Table, Materials and Methods, Figures, Data Availability Section)
If your work benefited from core facilities, was their service mentioned in the acknowledgments section?	Not Applicable	

Design

Study protocol	Information included in the manuscript?	In which section is the information available? (Reagents and Tools Table, Materials and Methods, Figures, Data Availability Section)
If study protocol has been **pre-registered, provide DOI in the manuscript**. For clinical trials, provide the trial registration number **OR** cite DOI.	Not Applicable	
Report the **clinical trial registration number** (at ClinicalTrials.gov or equivalent), where applicable.	Not Applicable	

Laboratory protocol	Information included in the manuscript?	In which section is the information available? (Reagents and Tools Table, Materials and Methods, Figures, Data Availability Section)
Provide DOI OR other citation details if **external detailed step-by-step protocols** are available.	Yes	Materials and methods; References section

Experimental study design and statistics	Information included in the manuscript?	In which section is the information available? (Reagents and Tools Table, Materials and Methods, Figures, Data Availability Section)
Include a statement about **sample size** estimate even if no statistical methods were used.	Yes	Material and Methods
Were any steps taken to minimize the effects of subjective bias when allocating animals/samples to treatment (e.g. **randomization procedure**)? If yes, have they been described?	Not Applicable	
Include a statement about **blinding** even if no blinding was done.	Not Applicable	
Describe **inclusion/exclusion criteria** if samples or animals were excluded from the analysis. Were the criteria pre-established?<br><br>If sample or data points were omitted from analysis, report if this was due to attrition or intentional exclusion and provide justification.	Yes	Results Section
For every figure, are **statistical tests** justified as appropriate? Do the data meet the assumptions of the tests (e.g., normal distribution)? Describe any methods used to assess it. Is there an estimate of variation within each group of data? Is the variance similar between the groups that are being statistically compared?	Yes	Results Section and Figure Captions

Sample definition and in-laboratory replication	Information included in the manuscript?	In which section is the information available? (Reagents and Tools Table, Materials and Methods, Figures, Data Availability Section)
In the figure legends: state number of times the experiment was **replicated** in laboratory.	Yes	Results Section and Figure Captions
In the figure legends: define whether data describe **technical or biological replicates**.	Yes	Results Section and Figure Captions

Ethics

Ethics	Information included in the manuscript?	In which section is the information available? (Reagents and Tools Table, Materials and Methods, Figures, Data Availability Section)
Studies involving **human participants**: State details of **authority granting ethics approval** (IRB or equivalent committee(s), provide reference number for approval.	Not Applicable	
Studies involving **human participants**: Include a statement confirming that **informed consent** was obtained from all subjects and that the experiments conformed to the principles set out in the WMA Declaration of Helsinki and the Department of Health and Human Services Belmont Report.	Not Applicable	
Studies involving **human participants:** For publication of **patient photos**, include a statement confirming that consent to publish was obtained.	Not Applicable	
Studies involving experimental **animals**: State details of **authority granting ethics approva**l (IRB or equivalent committee(s), provide reference number for approval. Include a statement of compliance with ethical regulations.	Not Applicable	
Studies involving **specimen and field samples:** State if relevant **permits** obtained, provide details of authority approving study; if none were required, explain why.	Not Applicable	

Dual Use Research of Concern (DURC)	Information included in the manuscript?	In which section is the information available? (Reagents and Tools Table, Materials and Methods, Figures, Data Availability Section)
Could your study fall under dual use research restrictions? Please check biosecurity documents and list of **select agents and toxins** (CDC): https://www.selectagents.gov/sat/list.htm	Not Applicable	
If you used a select agent, is the security level of the lab appropriate and reported in the manuscript?	Not Applicable	
If a study is subject to dual use research of concern regulations, is the name of the **authority granting approval and reference number** for the regulatory approval provided in the manuscript?	Not Applicable	

Reporting

The MDAR framework recommends adoption of discipline-specific guidelines, established and endorsed through community initiatives. Journals have their own policy about requiring specific guidelines and recommendations to complement MDAR.

Adherence to community standards	Information included in the manuscript?	In which section is the information available? (Reagents and Tools Table, Materials and Methods, Figures, Data Availability Section)
State if relevant guidelines or checklists (e.g., **ICMJE, MIBBI, ARRIVE, PRISMA**) have been followed or provided.	Not Applicable	
For **tumor marker prognostic studies,** we recommend that you follow the **REMARK** reporting guidelines (see link list at top right). See author guidelines, under 'Reporting Guidelines'. Please confirm you have followed these guidelines.	Not Applicable	
For **phase II and III randomized controlled trials**, please refer to the **CONSORT** flow diagram (see link list at top right) and submit the CONSORT checklist (see link list at top right) with your submission. See author guidelines, under 'Reporting Guidelines'. Please confirm you have submitted this list.	Not Applicable	

Data Availability

Data availability	Information included in the manuscript?	In which section is the information available? (Reagents and Tools Table, Materials and Methods, Figures, Data Availability Section)
Have **primary datasets** been deposited according to the journal's guidelines (see 'Data Deposition' section) and the respective accession numbers provided in the Data Availability Section?	Yes	Data Availability Section
Were **human clinical and genomic datasets** deposited in a public access-controlled repository in accordance to ethical obligations to the patients and to the applicable consent agreement?	Not Applicable	
Are **computational models** that are central and integral to a study available without restrictions in a machine-readable form? Were the relevant accession numbers or links provided?	Yes	Data Availability Section
If publicly available data were reused, provide the respective **data citations in the reference list**.	Yes	Materials and Methods; Reference Section
